# Rhodium-Catalyzed Arene Alkenylation: Selectivity and Reaction Mechanism as a Function of In Situ Oxidant Identity

**DOI:** 10.1021/acs.organomet.4c00327

**Published:** 2024-09-12

**Authors:** Marc T. Bennett, Kwanwoo A. Park, T. Brent Gunnoe

**Affiliations:** Department of Chemistry, University of Virginia, Charlottesville, Virginia 22904, United States

## Abstract

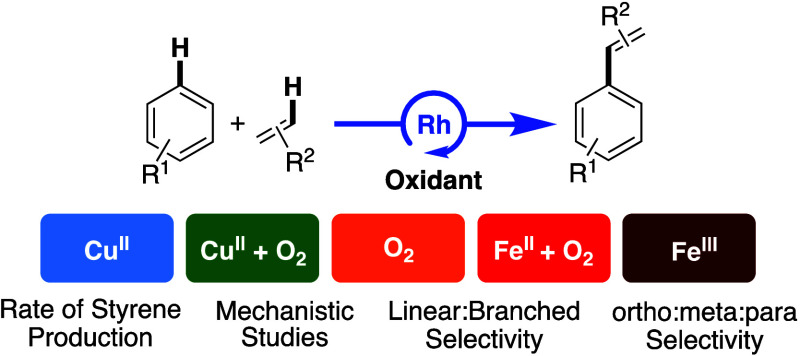

Rhodium catalyzed arene alkenylation reactions with arenes and olefins using dioxygen as the direct oxidant (e.g., *ACS Catal.***2020**, *10*, 11519), Cu(II) carboxylates (e.g., *Science***2015**, *348*, 421; *J. Am. Chem. Soc.***2017**, *139*, 5474) or Fe(III) carboxylate clusters (e.g., *ACS Catal.***2024,***14*, 10295), in the presence or absence of dioxygen, have been reported. These processes involve heating catalyst precursor [(η^2^-C_2_H_4_)_2_Rh(μ-OAc)]_2_, olefin, arene, and oxidant at temperatures between 120 and 200 °C. Herein, we report comparative studies of Rh-catalyzed arene alkenylation as a function of oxidant identity. This work includes comparisons of catalysis using Cu(II) carboxylates in the presence and absence of dioxygen, catalysis with only dioxygen as the oxidant, and Fe(III) carboxylates in the presence and absence of dioxygen. We report studies of catalysis with each oxidant including reagent concentration dependencies and kinetic isotope effect experiments using C_6_H_6_ or C_6_D_6_ and protio- or deutero carboxylic acid. Additionally, we probe ortho/meta/para regioselectivity for reactions of ethylene with monosubstituted arenes and Markovnikov/anti-Markovnikov selectivity with monosubstituted olefins. These studies indicate that the variation of oxidant identity impacts catalyst speciation, the reaction mechanism, and the reaction rate. Consequently, distinct Markovnikov/anti-Markovnikov and ortho/meta/para selectivities are observed for catalysis with each oxidant.

## Introduction

**Scheme 1:**
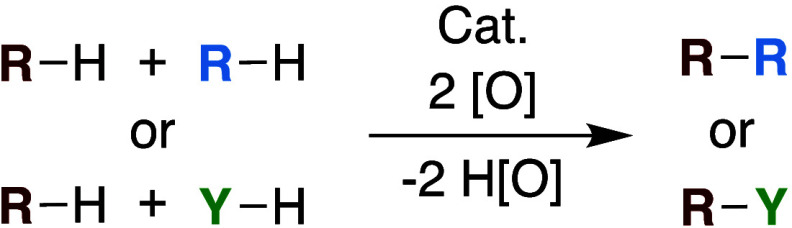
Reaction Scheme for Hydrocarbon Oxidative Functionalization Reactions (Y = Heteroatom)

Transition metal-catalyzed hydrocarbon oxidative functionalization reactions have been extensively studied over the last several decades.^1−11^ These processes often involve the formation of carbon–carbon or carbon–heteroatom bonds from substrates bearing carbon–hydrogen or heteroatom-hydrogen bonds, resulting in the net removal of two hydrogen atoms (Scheme 1). For oxidative functionalization processes, oxidation of hydrogen atoms is often required for a thermodynamically favorable reaction since the release of dihydrogen is often endothermic.^8^

**Scheme 2:**
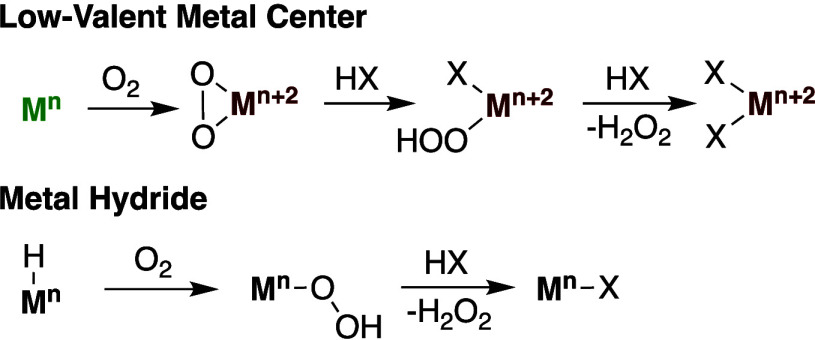
General Reaction Pathways of Low-Valent Metals or Metal Hydrides Reacting with Dioxygen When It Is Used as the Oxidant for Transition Metal Catalyzed Hydrocarbon Oxidative Functionalization Reactions

Given its abundance, dioxygen represents a potentially desirable oxidant for C–H oxidative functionalization reactions.^12,13^ Additionally, use of dioxygen as the ultimate oxidant is often necessary in order for large-scale oxidative functionalization processes to achieve economic viability.^14^ For some transition metal-catalyzed oxidative functionalization reactions, dioxygen has been reported as a suitable direct oxidant.^15−21^ Dioxygen is often proposed to react with low-valent metal centers (M^*n*^) to form M^*n*+2^(O_2_) intermediates or by net insertion into M–H intermediates to form an M–OOH intermediate (Scheme 2).^21−26^ In these reactions, the formed hydroperoxo or peroxo intermediates often react with protons generated earlier in the reaction,^27−30^ resulting in the release of H_2_O_2_, which can decompose to water.

**Scheme 3:**
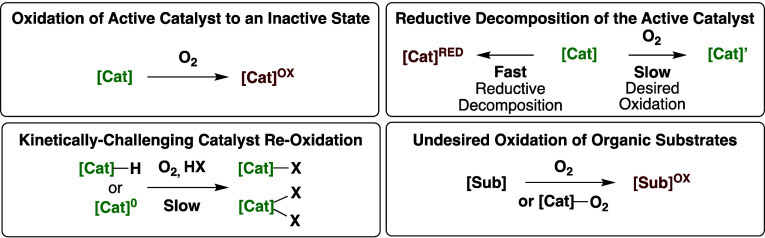
Challenges Associated with Use of Dioxygen as the In Situ Oxidant for Transition Metal Catalyzed Hydrocarbon Oxidative Functionalization Reactions

There are challenges associated with the use of dioxygen as the in situ oxidant for transition metal-catalyzed oxidative functionalization reactions (Scheme 3), including (1) kinetically challenging and/or unselective oxidation of low-valent metal centers or metal hydrides (resulting in a slow reaction rate),^15,26^ (2) low-valent metal centers can undergo reaction with dioxygen to form off-cycle species,^30−35^ (3) competitive reduction of an active catalyst to an inactive species (e.g., elemental Rh, Pt, or Pd) as a result of slow oxidation reactions,^21,36−38^ and/or (4) undesirable oxidation of substrates or products by dioxygen or by reactive metal-based intermediates formed upon reaction with dioxygen.^15,22^

**Scheme 4:**
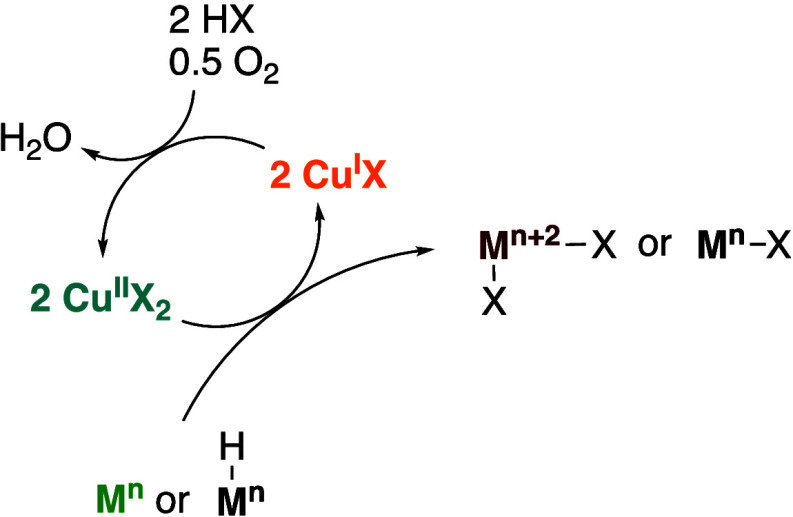
Demonstration of the Use of Cu(II) Salts as a Co-oxidant for Aerobic Hydrocarbon Oxidative Functionalization

As a result of the challenges associated with using dioxygen as the in situ oxidant, the use of dioxygen-recyclable co-oxidants that serve as the direct oxidant has been studied. An example of this strategy is the use of CuCl_2_ as a co-oxidant for ethylene Hoechst-Wacker oxidation.^39,40^ CuCl and HCl formed from CuCl_2_ can subsequently be reoxidized to CuCl_2_ by reaction with dioxygen, either during the reaction or in a separate step from catalysis.^40^ The strategy of using Cu(II) salts as oxidants has been widely employed for transition metal-catalyzed hydrocarbon oxidative functionalization reactions (Scheme 4).^1,7,40,41^

**Scheme 5:**
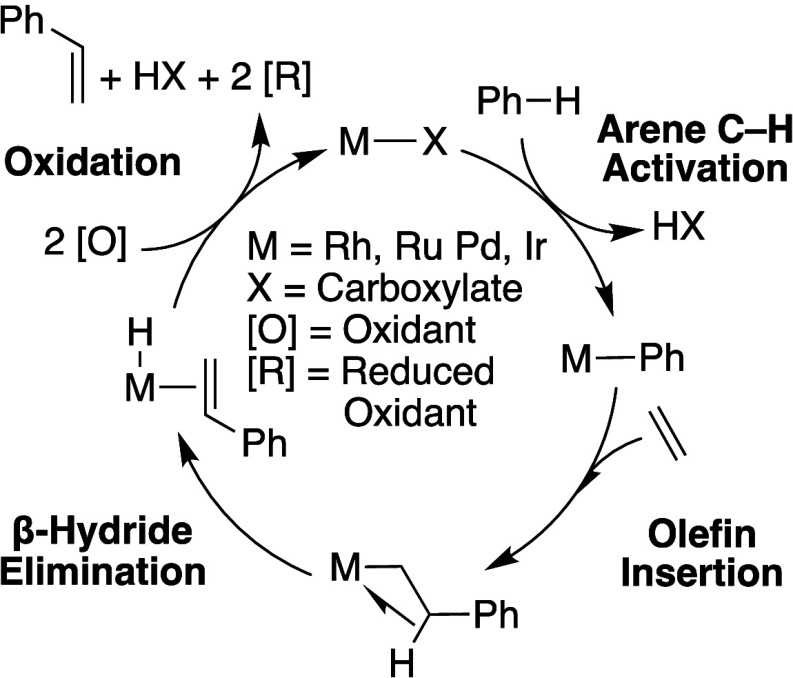
General Mechanism of Transition Metal Catalyzed Oxidative Arene Alkenylation

Transition metal-catalyzed arene alkylation or alkenylation reactions are of interest for large-scale petrochemical processes.^42−55^ Our group and others have studied Ru,^56^ Rh,^6,15,31,41,57−65^ Pd^57,65−67^ and Ir^68^ catalysts for the oxidative coupling of arenes and olefins to form alkenyl arenes (Scheme 5). These catalysts are usually proposed to operate through arene C–H activation, olefin insertion into the formed metal–aryl bond, β–hydride elimination, and oxidation of the M–H intermediate by two oxidizing equivalents.

**Scheme 6:**
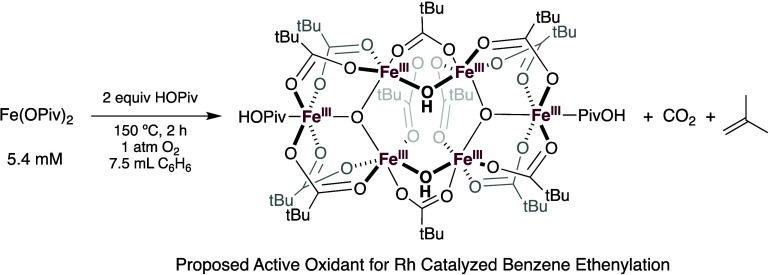
Formation of Fe_6_(μ-OH)_2_(μ_3_-O)_2_(μ-OPiv)_12_(HOPiv)_2_ from Fe(OPiv)_2_, HOPiv and Dioxygen Reproduced from ref (74) with permission.

Examples of arene-olefin oxidative coupling using stoichiometric oxidants such as AgOAc,^69^ organic peroxides,^70,71^ and ethylene^72,73^ have been reported. For Rh-, Pd-, and Ir-catalyzed styrene production from benzene and ethylene, our group and others have reported the use of Cu(II) carboxylate salts as an oxidant both in the presence and in the absence of dioxygen.^5,6,31,41,57−59,61−64,66−68^ Also, we reported the use of dioxygen as the sole oxidant for Rh-catalyzed styrene production from benzene and ethylene.^15^ Recently, we reported the use of Fe(II) carboxylate additives for aerobic Rh-catalyzed styrene production from benzene and ethylene.^74^ It was found that Fe(II) carboxylates and dioxygen react to form hexanuclear complex Fe_6_(μ-OH)_2_(μ_3_-O)_2_(μ-X)_12_(HX)_2_ (X = carboxylate), which is the likely active oxidant. Fe_6_(μ-OH)_2_(μ_3_-O)_2_(μ-OPiv)_12_(HOPiv)_2_ was proposed to form from Fe(OPiv)_2_ by a mechanism that involves abstraction of two H atoms from HOPiv by an oxidative decarboxylation mechanism (Scheme 6). Fe_6_(μ-OH)_2_(μ_3_-O)_2_(μ-X)_12_(HX)_2_ is also active as an oxidant under anaerobic conditions.

**Scheme 7:**
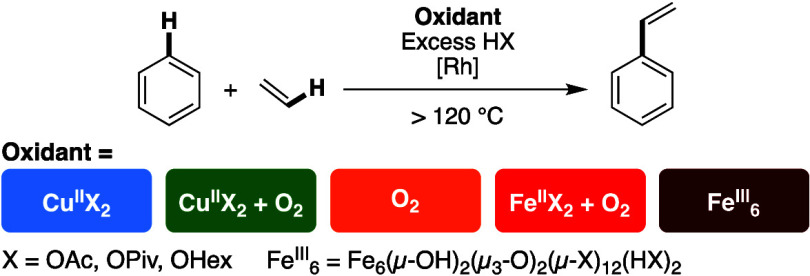
Oxidant Systems Studied Previously by Our Laboratory for Rh Catalyzed Styrene Production from Benzene and Ethylene Comparative studies of these five oxidant systems are undertaken herein.

Our group has observed styrene production from benzene and ethylene using [(η^2^-C_2_H_4_)_2_Rh(μ-OAc)]_2_ as the catalyst precursor with an excess of carboxylic acid using each of the five following oxidant systems (Scheme 7): (1) Cu(II) carboxylates under anaerobic conditions (**Cu**^**II**^) (2) Cu(II) carboxylates in the presence of dioxygen (**Cu**^**II**^**/O**_**2**_), (3) only dioxygen (**O**_**2**_), (4) FeX_2_ (X = carboxylate) in the presence of dioxygen (**Fe**^**II**^**/O**_**2**_), and (5) Fe_6_(μ-OH)_2_(μ_3_-O)_2_(μ-X)_12_(HX)_2_ in the absence of dioxygen (**Fe**^**III**^). With the goal of understanding the role of oxidants in the catalytic cycle and the effect of dioxygen on catalysis, we sought to compare these five oxidant systems.

Variation of the oxidant system for Rh-catalyzed arene alkenylation can change the kinetics of Rh–H oxidation, which can impact the overall reaction rate. Beyond influencing the oxidation step, the oxidant identity could influence catalyst speciation. As an example, previously our laboratory found that catalyst precursor [(η^2^-C_2_H_4_)_2_Rh(μ-OAc)]_2_ reacts with Cu(OPiv)_2_ to form [(η^2^-C_2_H_4_)_2_Rh(μ-OPiv)]_2_(μ-Cu), which was proposed to be an active catalyst.^59^ Density functional theory (DFT) calculations found that the inclusion of Cu(II) in the active catalyst results in lower activation barriers than those for catalysts lacking Cu(II) atoms. Similarly, for catalysis with Pd(OAc)_2_ as the catalyst precursor, we identified the complex PdCu_2_(μ-OPiv)_6_ as a likely active catalyst.^66^ For both Rh and Pd catalysis, orders in catalyst concentration between one and two were observed likely as a result of a complicated equilibrium between active mono- and bis-Rh or Pd complexes that contain Cu(II) atoms.^57^ Other groups have invoked similar heterometallic complexes that incorporate Cu(II) or Ag(I) for oxidative coupling reactions.^75−77^ Also, the presence of an oxidant can lead to undesired oxidation of an active Rh(I) species to inactive (off-cycle) intermediates. For example, we observed a small inverse dependence on Cu(OPiv)_2_ concentration in a previous study, potentially suggesting that Cu(OPiv)_2_ might oxidize Rh to inactive intermediates.^64^ Also, we reported a decrease in reaction rate upon the addition of dioxygen to a reaction using Cu(OPiv)_2_ as the oxidant, suggesting that dioxygen might react with Rh to form off-cycle intermediates.^31^ These findings indicate that identification of an oxidant and reaction conditions at which the desired Rh–H oxidation occurs rapidly and selectively, while undesired catalyst oxidation does not occur, is needed for a successful catalytic system. When metal-based co-oxidants are utilized, heterometallic complexes can form, which can enhance catalyst activity.

Herein, we report comparative studies of Rh-catalyzed arene alkenylation as a function of oxidant identity, including selectivity for alkenyl arenes versus undesired side products, kinetic isotope effect (KIE) experiments, and reagent concentration effects. The effect of oxidant identity on arene C–H activation regioselectivity was probed by studying the ortho/meta/para regioselectivity of arene ethenylation using anisole, *tert-*butylbenzene, toluene, chlorobenzene, and α,α,α-trifluorotoluene as the arene substrates. To probe for differences in the C–H activation mechanism, kinetic intermolecular competition experiments between toluene and α,α,α-trifluorotoluene were performed. Anti-Markovnikov versus Markovnikov selectivity and selectivity for internal versus terminal alkenyl products were studied using propylene as the olefin, and the studies were extended to *tert-*butyl ethylene, methyl acrylate, and styrene.

## Results and Discussion

### Rate of Styrene Production and Selectivity for Styrene

The kinetics of benzene ethenylation at 150 °C were probed to quantify differences in the reaction rate for catalysis with each of the oxidant combinations. Each of these reactions involved the combination of benzene (7.5 mL), a 0.001 mol % loading (relative to benzene per single Rh atom) of the catalyst precursor [(η^2^-C_2_H_4_)_2_Rh(μ-OAc)]_2_, 960 equiv of HOPiv (relative to Rh) (OPiv = trimethyl acetate), and 70 psig of ethylene. For catalysis with each of the oxidants, the following components were added: **Cu**^**II**^: 480 equiv of Cu(OPiv)_2_, **Cu**^**II**^**/O**_**2**_: 480 equiv of Cu(OPiv)_2_ and 1 atm of dioxygen, **O**_**2**_: 1 atm of dioxygen, **Fe**^**II**^**/O**_**2**_: 480 equiv of Fe(OAc)_2_ and 1 atm O_2_, **Fe**^**III**^: 80 equiv of Fe_6_(μ-OH)_2_(μ_3_-O)_2_(μ-X)_12_(HX)_2_. We note that for **Cu**^**II**^ catalysis since two equiv of Cu(OPiv)_2_ are required for each turnover of styrene, the maximum number of turnovers is 240 since 480 equiv of Cu(OPiv)_2_ are used. Likewise, for catalysis with **Fe**^**III**^, each hexanuclear cluster serves as one oxidizing equiv, so the maximum number of turnovers is 40 since 80 equiv of Fe_6_(μ-OH)_2_(μ_3_-O)_2_(μ-X)_12_(HX)_2_ are used. For the **Cu**^**II**^**/O**_**2**_ and **Fe**^**II**^**/O**_**2**_ systems, in situ aerobic reoxidation has been demonstrated, so there is not a limiting quantity of oxidizing Cu or Fe equivalents.

**Figure 1:**
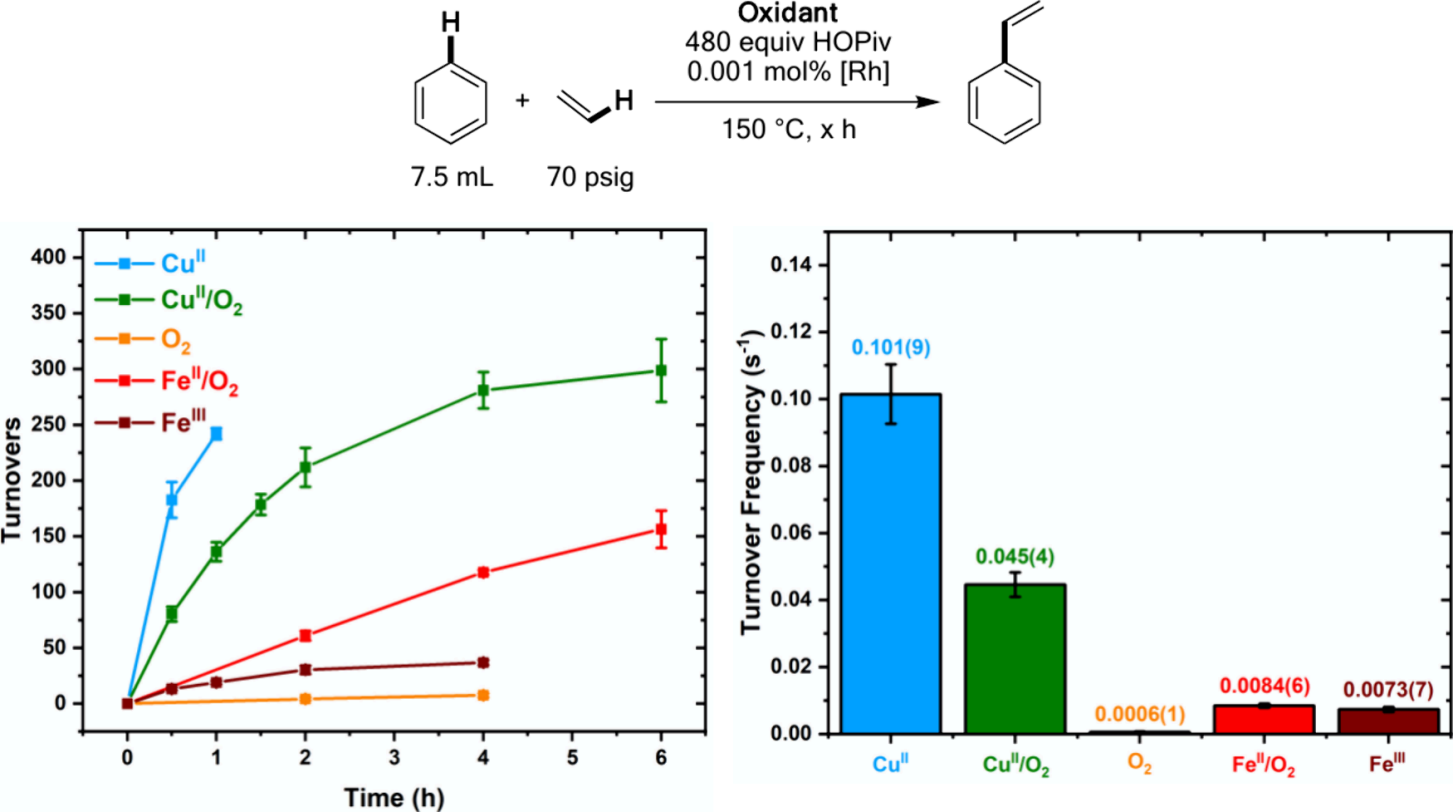
Kinetics of benzene ethenylation at 150 °C. Reaction conditions: 7.5 mL of benzene, 0.001 mol % (relative to benzene per single Rh atom) [(η^2^-C_2_H_4_)_2_Rh(μ-OAc)]_2_, 960 equiv HOPiv (relative to Rh), 70 psig ethylene, and the following for each oxidant system: **Cu**^**II**^: 480 equiv Cu(OPiv)_2_, **Cu**^**II**^**/O**_**2**_: 480 equiv Cu(OPiv)_2_ and 1 atm O_2_, **O**_**2**_: 1 atm O_2_, **Fe**^**II**^**/O**_**2**_: 480 equiv Fe(OAc)_2_ and 1 atm O_2_, **Fe**^**III**^: 80 equiv Fe_6_(μ-OH)_2_(μ_3_-O)_2_(μ-OPiv)_12_(HOPiv)_2_ 150 °C. The turnover frequencies shown on the right were calculated based on TOs at the first recorded data point. Each data point represents the average of a minimum of three independent experiments, and the error bars represent the standard deviation from the multiple experiments (a minimum of three).

As shown in Figure 1, **Cu**^**II**^ catalysis results in an initial turnover frequency of 0.101(9) s^–1^ based on the rate of styrene formation between 0 and 0.5 h, and the catalysis likely achieves ∼240 turnovers prior to the 1-h time point. Catalysis using **Cu**^**II**^**/O**_**2**_ results in an initial (based on the 0.5 h time point) turnover frequency of 0.029(2) s^–1^, ∼3-fold slower than an otherwise identical reaction using only **Cu**^**II**^. For **O**_**2**_, an initial turnover frequency of ∼0.0006(1) s^–1^ was observed. **Fe**^**II**^**/O**_**2**_ yields an initial turnover frequency of 0.0084(6) s^–1^, and a linear plot of turnovers versus time was observed for the 6 h course of the reaction. The turnover frequency with **Fe**^**II**^**/O**_**2**_ is slower than that observed for **Cu**^**II**^**/O**_**2**_, suggesting either the active catalyst for **Fe**^**II**^**/O**_**2**_ is less active than that formed for **Cu**^**II**^**/O**_**2**_ or that the proposed active oxidant for **Fe**^**II**^**/O**_**2**_, Fe_6_(μ-OH)_2_(μ_3_-O)_2_(μ-OPiv)_12_(HOPiv)_2_, reacts more slowly with Rh–H intermediates than does Cu(OPiv)_2_. This will be discussed in more detail below. The use of **Fe**^**III**^ results in an initial turnover frequency only slightly slower than that observed for **Fe**^**II**^**/O**_**2**_, suggesting no significant change in catalyst speciation, mechanism, or activity upon the addition of dioxygen. As noted above, 80 equiv of Fe_6_(μ-OH)_2_(μ_3_-O)_2_(μ-OPiv)_12_(HOPiv)_2_ is expected to form a maximum of 40 TOs of styrene since each hexanuclear cluster serves as one oxidizing equivalent. Also, the rate for **Fe**^**III**^ catalysis decreases as the reaction progresses, which can be attributed to the reaction’s first-order dependence on Fe_6_(μ-OH)_2_(μ_3_-O)_2_(μ-OPiv)_12_(HOPiv)_2_ concentration.^74^ The similarity in initial turnover frequency for **Fe**^**II**^**/O**_**2**_ and **Fe**^**III**^ contrasts with the case of **Cu**^**II**^**/O**_**2**_ and **Cu**^**II**^, for which a substantially slower turnover frequency is observed for **Cu**^**II**^**/O**_**2**_**.** These findings potentially suggest that the active species for **Fe**^**II**^**/O**_**2**_ and **Fe**^**III**^ catalysis is less sensitive to dioxygen than that for **Cu**^**II**^**/O**_**2**_ and **Cu**^**II**^ catalysis.

**Figure 2:**
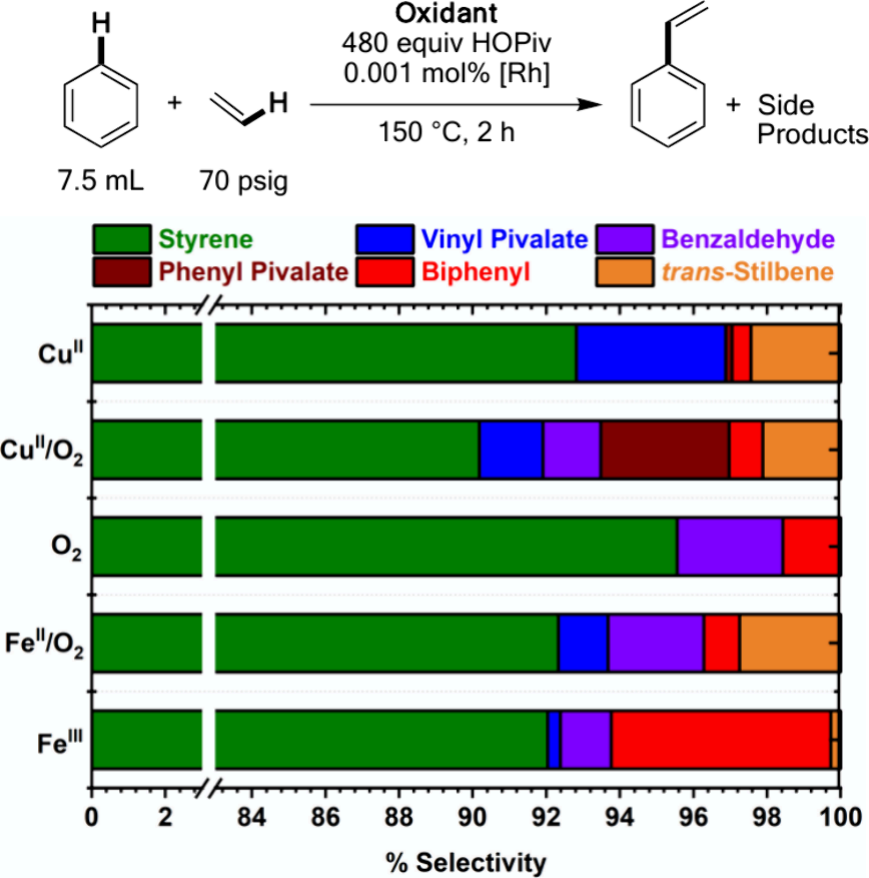
Selectivity of benzene ethenylation at 150 °C. Reaction conditions: 7.5 mL benzene, 0.001 mol % (relative to benzene per single Rh atom) [(η^2^-C_2_H_4_)_2_Rh(μ-OAc)]_2_, 960 equiv HOPiv (relative to Rh), 70 psig ethylene, and the following for each oxidant system: **Cu**^**II**^: 480 equiv Cu(OPiv)_2_, **Cu**^**II**^**/O**_**2**_: 480 equiv Cu(OPiv)_2_ and 1 atm O_2_, **O**_**2**_: 1 atm O_2_, **Fe**^**II**^**/O**_**2**_: 480 equiv Fe(OAc)_2_ and 1 atm O_2_, **Fe**^**III**^: 80 equiv Fe_6_(μ-OH)_2_(μ_3_-O)_2_(μ-OPiv)_12_(HOPiv)_2_ 150 °C, 2 h. Each data point represents the average of three independent experiments.

For the reactions shown in Figure 1, we next probed the selectivity for styrene versus undesired side products at the 2 h time point, or 1 h for **Cu**^**II**^ since the reaction is likely to complete prior to 1 h. As shown in Figure 2, **Cu**^**II**^ catalysis gives ∼93% selectivity for styrene, with vinyl pivalate and *trans*-stilbene representing the most significant side products. Also, minor amounts of biphenyl and phenyl pivalate were observed. Previously, our group studied Cu(II) carboxylate-mediated phenyl ester production and found that the reaction is kinetically slow at 150 °C.^78^ The lack of significant phenyl ester production is likely the result of both the slow rate of phenyl ester formation at 150 °C and the comparatively rapid rate of styrene production, which consumes Cu(OPiv)_2_. The production of *trans*-stilbene, which forms as a result of styrene undergoing oxidative hydrophenylation, can likely be avoided by the use of higher ethylene pressure or by continuous removal of styrene in a more complex reactor design. For **Cu**^**II**^**/O**_**2**_ catalysis, benzaldehyde is formed as a side product, forming as the result of styrene undergoing reaction with dioxygen and/or perhaps with reactive Rh- or Cu-dioxygen intermediates. More phenyl pivalate is observed for **Cu**^**II**^**/O**_**2**_ than for **Cu**^**II**^, which is possibly attributable to the extended reaction time and comparatively slower rate of Rh catalysis for **Cu**^**II**^**/O**_**2**_ versus **Cu**^**II**^. Similar quantities of biphenyl and *trans*-stilbene side products were observed between **Cu**^**II**^ and **Cu**^**II**^**/O**_**2**_. For catalysis with **O**_**2**_, ∼ 95% selectivity for styrene is observed with benzaldehyde and biphenyl representing the only significant side products. The lack of significant *trans*-stilbene using **O**_**2**_ is likely the result of the low concentration of styrene that forms due to the slow reaction rate; thus, ethylene is present in a larger excess relative to styrene in comparison to other oxidant systems. **Fe**^**II**^**/O**_**2**_ catalysis yields approximately 92% selectivity for styrene, with moderate quantities of all side products except for phenyl pivalate/acetate, which is not produced in detectable quantities. For **Fe**^**III**^, benzaldehyde is produced as a side product, suggesting that Fe_6_(μ-OH)_2_(μ_3_-O)_2_(μ-OPiv)_12_(HOPiv)_2_ is capable of mediating styrene oxidation in the absence of dioxygen. Biphenyl is observed as a more significant side product for **Fe**^**III**^ than for **Fe**^**II**^**/O**_**2**_. Similar to the case with **O**_**2**_, since only 40 TOs of styrene are produced for **Fe**^**III**^ catalysis, styrene does not reach a sufficient concentration to significantly compete with ethylene as an olefin substrate, so only trace quantities of *trans*-stilbene are observed.

### Mechanistic Studies of Styrene Production

**Scheme 8:**
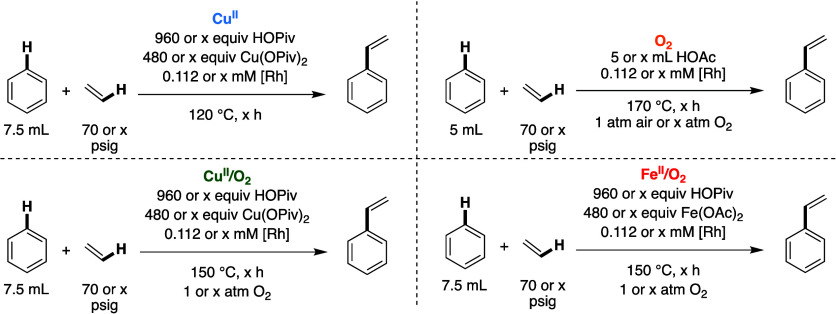
Overview of Reaction Conditions Employed for Studies of Reaction Rate Dependencies For **Cu**^**II**^, **Cu**^**II**^**/O**_**2**_ and **Fe**^**II**^**/O**_**2**_, [Rh] = [(η^2^-C_2_H_4_)_2_Rh(μ-OAc)]_2_ (loading is based on single Rh atom), and for **O**_**2**_, [Rh] = RhCl_3_ (loading is relative to single Rh atom).

In this section, we combine results from previously published mechanistic studies of **O**_**2**_(15) and **Fe**^**II**^**/O**_**2**_(74) catalysis with new studies of **Cu**^**II**^ and **Cu**^**II**^**/O**_**2**_ systems. The reaction conditions employed for the studies are listed in Scheme 8. Studies of **Cu**^**II**^ were carried out at 120 °C because the turnover frequency is sufficiently fast at 150 °C that reliably recording three-time points was not feasible. **Cu**^**II**^**/O**_**2**_ and previously published **Fe**^**II**^**/O**_**2**_ reactions were performed at 150 °C,^74^ and **O**_**2**_ reactions, which are previously published,^15^ were performed at 170 °C because the reaction is slow at 150 °C. For reagent concentration dependence studies in this section, we display log–log plots for the four oxidant systems on one graph, but the *k*_obs_ values cannot be meaningfully compared as different reaction temperatures were employed. For comparisons of reaction rates for the five systems at identical conditions, see Figure 1 above. Mechanistic studies were not carried out for **Fe**^**III**^ because our results herein indicate similar reaction rates and selectivity for **Fe**^**III**^ and **Fe**^**II**^**/O**_**2**_, suggesting similar processes whether dioxygen is used in situ or not.

### Kinetic Isotope Effect Studies

**Figure 3:**
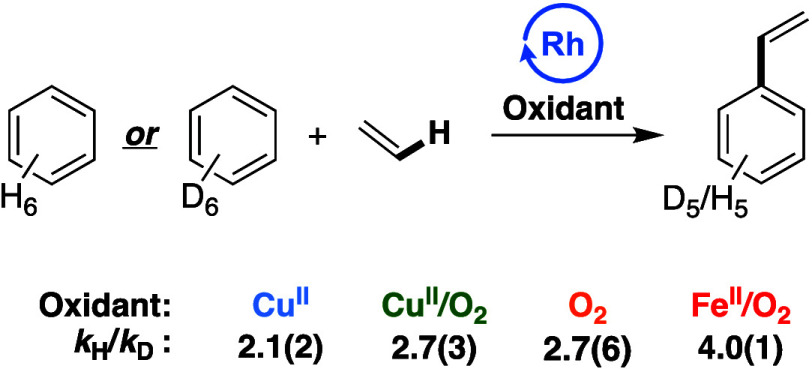
KIE experiments utilizing benzene or benzene-*d*_6_ in parallel independently performed reactions. Reaction conditions: **Cu**: 7.5 mL of benzene or benzene-*d*_6_, 70 psig of ethylene, 0.112 mM (per single Rh atom) [(η^2^-C_2_H_4_)_2_Rh(μ-OAc)]_2_, 480 equiv of Cu(OPiv)_2_, 960 equiv of HOPiv, 120 °C. **Cu/O**_**2**_: 7.5 mL of benzene or benzene-*d*_6_, 70 psig ethylene, 0.112 mM (based on single Rh atom) [(η^2^-C_2_H_4_)_2_Rh(μ-OAc)]_2_, 480 equiv Cu(OPiv)_2_, 960 equiv HOPiv, 1 atm O_2_, 150 °C. **O**_**2**_ (previously reported)^15^ 5 mL of benzene or benzene-*d*_6_, 70 psig ethylene, 0.112 mM RhCl_3_, 5 mL HOAc, 1 atm air, 170 °C. **Fe/O**_**2**_ (previously published):^74^ 7.5 mL of benzene or benzene-*d*_6_, 70 psig ethylene, 0.112 mM (based on single Rh atom) [(η^2^-C_2_H_4_)_2_Rh(μ-OAc)]_2_, 480 equiv Fe(OAc)_2_, 960 equiv HOPiv, 1 atm O_2_, 150 °C. Each KIE represents the average of a minimum of three independent experiments with the standard deviation from the multiple experiments shown in parentheses.

Kinetic isotope effect (KIE) experiments utilizing either C_6_H_6_ or C_6_D_6_ were carried out to determine the kinetic relevancy of arene C–H activation. Previously published^15,74^*k*_H_*/k*_D_ values for **O**_**2**_ and **Fe**^**II**^**/O**_**2**_ catalysis are shown in Figure 3, in addition to new data for **Cu**^**II**^ and **Cu**^**II**^**/O**_**2**_. As shown in Figure 3, primary KIEs between ∼2 and ∼4 were observed for the four oxidant systems, indicating (1) the likelihood of unique active catalysts and/or mechanisms for each of the processes and (2) kinetically relevant benzene C–H activation steps for each of the processes. Each of the KIE values is consistent with previously reported values for processes that operate through late transition metal-mediated C–H activation.^67,76,79,80^

### Carboxylic Acid Effects

**Figure 4:**
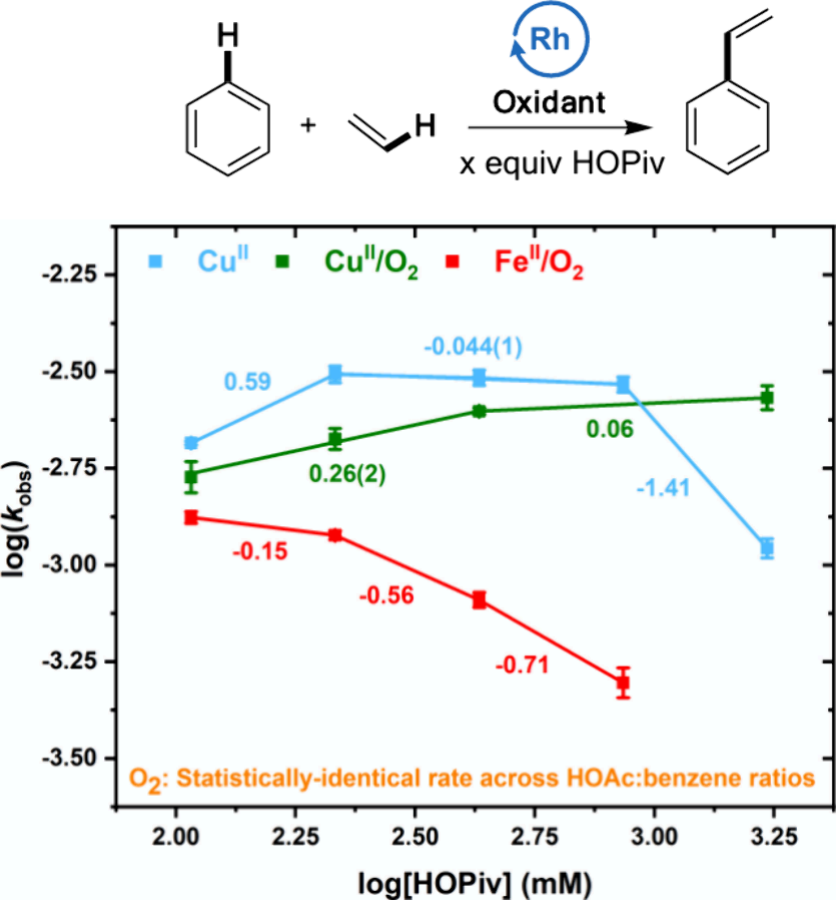
Log–log plots for the dependence of the reaction rate on the HOPiv concentration. The log[HOPiv] values represent the log of [HOPiv] in mM. **Cu**^**II**^: 7.5 mL of benzene, 70 psig of ethylene, 0.112 mM (based on single Rh atom) [(η^2^-C_2_H_4_)_2_Rh(μ-OAc)]_2_, 480 equiv of Cu(OPiv)_2_, x equiv of HOPiv, 120 °C. **Cu**^**II**^**/O**_**2**_: 7.5 mL of benzene, 70 psig ethylene, 0.112 mM (based on single Rh atom) [(η^2^-C_2_H_4_)_2_Rh(μ-OAc)]_2_, 480 equiv Cu(OPiv)_2_, x equiv HOPiv, 1 atm O_2_, 150 °C. **Fe**^**II**^**/O**_**2**_ (previously published):^74^ 7.5 mL of benzene, 70 psig ethylene, 0.112 mM (based on single Rh atom) [(η^2^-C_2_H_4_)_2_Rh(μ-OAc)]_2_, 480 equiv Fe(OAc)_2_, x equiv HOPiv, 1 atm O_2_, 150 °C. Previous studies of **O**_**2**_ indicate no significant change in rate as a function of the HOAc:benzene ratio.^15^ Each data point represents the average of a minimum of three independent experiments, and the error bars represent the standard deviations from multiple experiments.

To determine the effect of carboxylic acid concentration on the reaction rate, experiments with varying HOPiv concentrations were carried out (Figure 4). We note that the presence of HOPiv increases the solubility of Cu(II) and Fe(III) carboxylates, and at all conditions, the reaction samples appeared homogeneous, although the possibility of partial insolubility under some conditions cannot be excluded. Previously published results using **O**_**2**_ revealed statistically identical rates of reaction using benzene:HOAc volume:volume ratios from 9:1 to 1:9, indicating that the reaction with **O**_**2**_ is likely zero-order in HOAc. As shown in Figure 4, the effect of HOPiv concentration on the rate of styrene production was probed using **Cu**^**II**^, **Cu**^**II**^**/O**_**2**_, and **Fe**^**II**^**/O**_**2**_. We note that since nonlinearity is observed for each of the systems, orders cannot be calculated, but slopes are provided to give context to the observed dependencies. Catalysis using **Cu**^**II**^ gives an initial positive dependence on HOPiv as its concentration is increased. This positive dependence saturates, and the use of higher HOPiv concentrations results in a decrease in reaction rate. With **Cu**^**II**^**/O**_**2**_, an initial positive dependence on HOPiv is observed, which saturates at higher concentrations. The observation of a positive dependence on HOPiv at low concentrations is consistent with our group’s previous finding that carboxylic acid inhibits the reaction of Cu(I) carboxylate with water to form Cu oxides.^31^ It is also possible that the presence of HOPiv inhibits or reverses an undesired reaction of an active Rh(I) species with dioxygen to form off-cycle intermediates. This possibility is discussed in more detail below.

**Figure 5:**
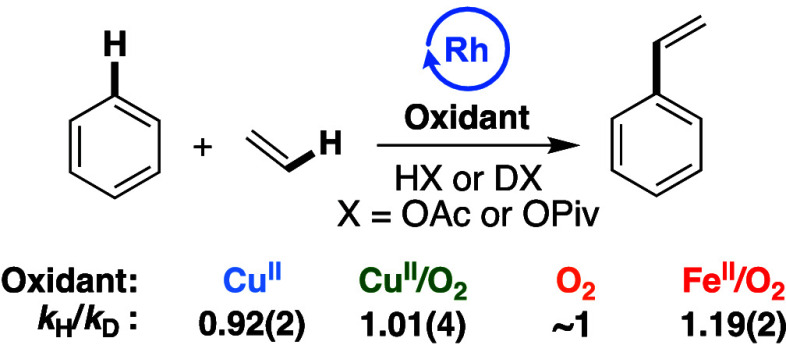
KIE experiments utilizing protio- or deutero-carboxylic acid. Reaction conditions: **Cu**^**II**^: 7.5 mL of benzene, 70 psig of ethylene, 0.112 mM (based on single Rh atom) [(η^2^-C_2_H_4_)_2_Rh(μ-OAc)]_2_, 480 equiv of Cu(OPiv)_2_, 960 equiv of HOPiv or DOPiv, 120 °C. **Cu**^**II**^**/O**_**2**_: 7.5 mL benzene, 70 psig ethylene, 0.112 mM (based on single Rh atom) [(η^2^-C_2_H_4_)_2_Rh(μ-OAc)]_2_, 480 equiv Cu(OPiv)_2_, 960 equiv HOPiv or DOPiv, 1 atm O_2_, 150 °C. **O**_**2**_ (previously reported):^15^ 5 mL of benzene, 70 psig ethylene, 0.112 mM RhCl_3_, 5 mL acetic acid or acetic acid-*d*_4_, 1 atm air, 170 °C. **Fe**^**II**^**/O**_**2**_ (previously published):^74^ 7.5 mL benzene, 70 psig ethylene, 0.112 mM (based on single Rh atom) [(η^2^-C_2_H_4_)_2_Rh(μ-OAc)]_2_, 480 equiv Fe(OAc)_2_, 960 equiv HOPiv or DOPiv, 1 atm O_2_, 150 °C. Each data point represents the average of a minimum of three independent experiments, and the standard deviations from the multiple experiments are shown in parentheses.

KIE experiments using either HOPiv or pivalic acid-*d*_1_ (DOPiv) for **Cu**^**II**^, **Cu**^**II**^**/O**_**2**_, and **Fe**^**II**^**/O**_**2**_ and HOAc or DOAc for **O**_**2**_ were carried out (Figure 5). The results indicate no significant difference in reaction rate between HOPiv and DOPiv with **Cu**^**II**^ or **Cu**^**II**^**/O**_**2**_, and previous results indicate no statistically significant KIE for **O**_**2**_ catalysis when acetic acid or acetic acid-*d*_*4*_ are utilized.^15^ Previously, we reported a small KIE of 1.19(2) for **Fe**^**II**^**/O**_**2**_ catalysis. We speculated that the small KIE for **Fe**^**II**^**/O**_**2**_ might be attributed to the protonation of an Rh–OH intermediate, which could form upon the reaction of two equiv of Fe_6_(μ-OH)_2_(μ_3_-O)_2_(μ-OPiv)_12_(HOPiv)_2_ with an Rh–H intermediate.^74^

**Scheme 9:**
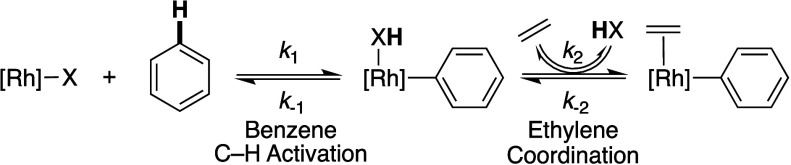
Proposed Mechanism of Arene C–H Activation and Ethylene Coordination for a Process In Which Benzene C–H Activation Is Reversible

For a reversible C–H activation step, increasing the concentration of carboxylic acid could influence the equilibrium between a Rh(HX)(Ph) intermediate and a Rh(C_2_H_4_) intermediate (Scheme 9). Additionally, decreasing the ethylene concentration could decrease the rate of ethylene coordination (*k*_2_[C_2_H_4_]), which could result in a longer-lived Rh(Ph)(HX) intermediate, resulting in an increased propensity for the reverse of C–H activation to occur.

**Figure 6:**
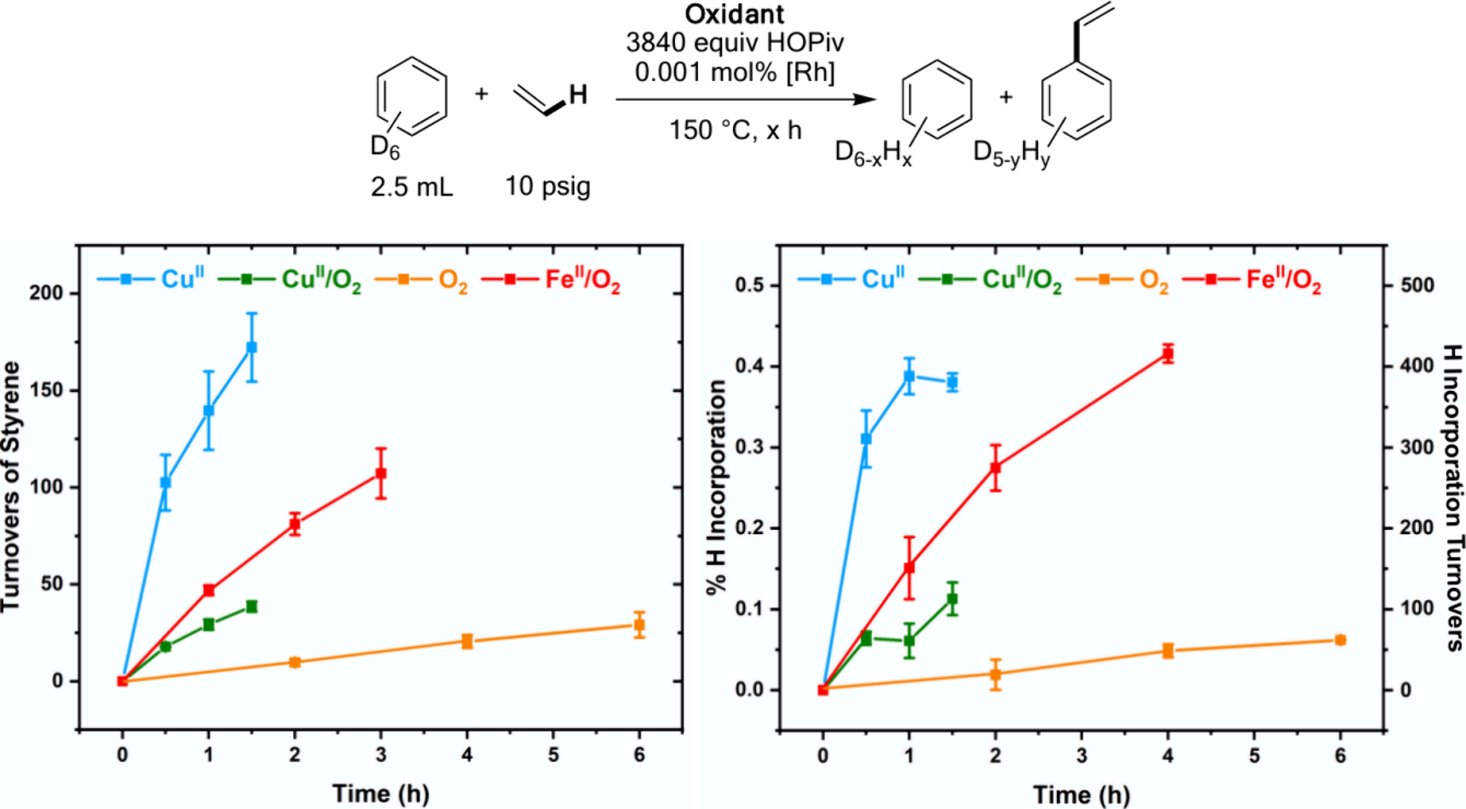
Studies of proton incorporation into C_6_D_6_ when using 10 psig of ethylene and 3840 equiv of HOPiv. Reaction conditions: 2.5 mL of C_6_D_6_, 0.001 mol % [(η^2^-C_2_H_4_)_2_Rh(μ-OAc)]_2_ (relative to C_6_D_6_ per single Rh atom), 3840 equiv (relative to single Rh atom) HOPiv, 10 psig of ethylene, and the following for each oxidant system: **Cu**^**II**^: 480 equiv Cu(OPiv)_2_, **Cu**^**II**^**/O**_**2**_: 480 equiv Cu(OPiv)_2_ and 1 atm O_2_, **O**_**2**_: 1 atm O_2_, **Fe**^**II**^**/O**_**2**_: 480 equiv Fe(OAc)_2_ and 1 atm O_2_. Each data point represents the average of a minimum of three independent experiments, and error bars represent the standard deviations for the multiple independent experiments.

Previously, we observed that the predilection toward reverse C–H activation occurring during **Cu**^**II**^ catalysis is dependent upon the relative concentrations of HOPiv and olefin.^58^ At high ethylene concentration and low HOPiv concentration, C–H activation was proposed to be minimally reversible, likely because the rate of ethylene coordination is rapid (i.e., *k*_2_[C_2_H_4_] > *k*_–2_[HOPiv]). Conversely, at high HOPiv concentration and low ethylene pressure, the C–H activation step had enhanced reversibility (*k*_2_[C_2_H_4_] < *k*_–2_[HOPiv]). We probed the reversibility of benzene C–H activation under reaction conditions in which the HOPiv concentration is high and the ethylene pressure is low with each of the oxidant systems to help determine if the observed [HOPiv] dependencies could originate from a reversible C–H activation. To do so, C_6_D_6_ was used as arene with 3840 equiv of HOPiv relative to Rh and 10 psig of ethylene. The incorporation of protons into C_6_D_6_ and the rate of styrene formation was monitored by GC–MS (Figure 6).

The results in Figure 6 indicate that arene C–H activation is reversible for **Cu**^**II**^, **Cu**^**II**^**/O**_**2**_**, O**_**2**_, and **Fe**^**II**^**/O**_**2**_ catalysis. Additionally, the relative rates of H/D exchange between the oxidants correlate with the relative rates of styrene formation, as shown in Figure 1 above. The inverse dependencies in the HOPiv concentration for **Cu**^**II**^ and **Fe**^**II**^**/O**_**2**_ catalysis (Figure 4) likely originate from HOPiv accelerating the rate of the reverse of C–H activation, which, in turn, decreases the rate of styrene formation. Interestingly, arene C–H activation is likely reversible for **Cu**^**II**^**/O**_**2**_ and **O**_**2**_ catalysis, yet increasing [HOPiv] does not decelerate the rate of styrene formation. Similar effects were observed for the ethylene pressure dependence for which **Cu**^**II**^ catalysis was inhibited by ethylene at high pressure, but **Cu**^**II**^**/O**_**2**_ and **O**_**2**_ catalysis was not (see below).

### Effect of Ethylene Concentration

**Figure 7:**
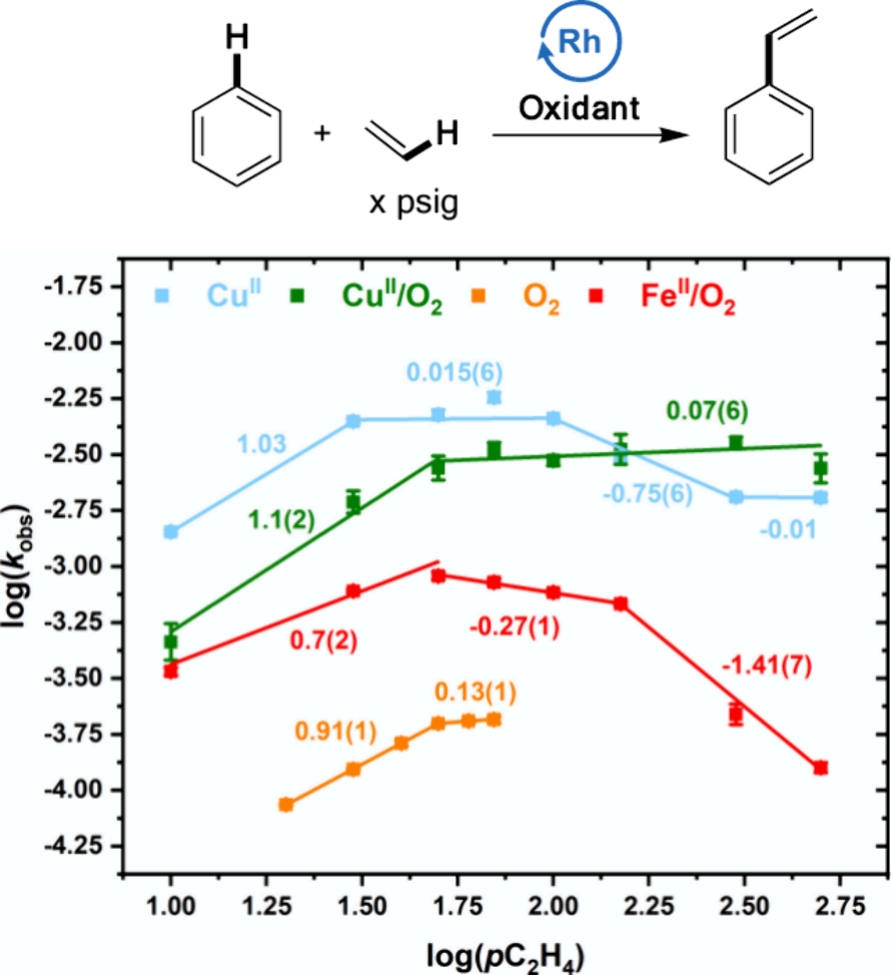
Log–log plots for the dependence of the reaction rate on ethylene pressure. Reaction conditions: **Cu**^**II**^: 7.5 mL of benzene, x psig of ethylene, 0.112 mM (based on single Rh atom) [(η^2^-C_2_H_4_)_2_Rh(μ-OAc)]_2_, 480 equiv of Cu(OPiv)_2_, 960 equiv of HOPiv, 120 °C. **Cu**^**II**^**/O**_**2**_: 7.5 mL of benzene, x psig ethylene, 0.112 mM (based on single Rh atom) [(η^2^-C_2_H_4_)_2_Rh(μ-OAc)]_2_, 480 equiv Cu(OPiv)_2_, 960 equiv HOPiv, 1 atm O_2_, 150 °C. **O**_**2**_ (previously reported):^15^ 5 mL of benzene, x psig ethylene, 0.112 mM RhCl_3_, 5 mL HOAc, 1 atm air, 170 °C. **Fe**^**II**^**/O**_**2**_ (previously published):^74^ 7.5 mL of benzene, x psig ethylene, 0.112 mM (based on single Rh atom) [(η^2^-C_2_H_4_)_2_Rh(μ-OAc)]_2_, 480 equiv Fe(OAc)_2_, 960 equiv HOPiv, 1 atm O_2_, 150 °C. Each data point represents the average of a minimum of three independent experiments, and the error bars represent the standard deviations from the multiple experiments.

**Scheme 10:**
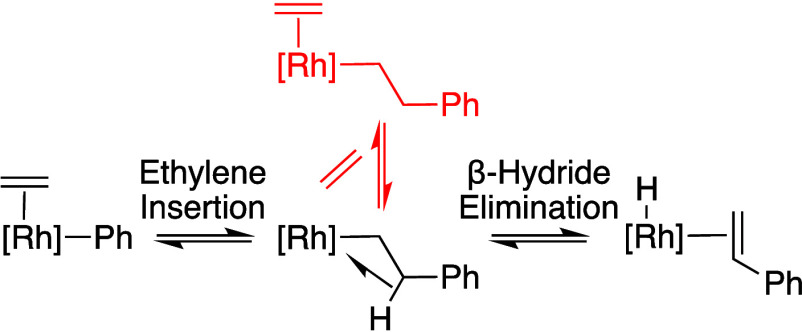
Proposed Coordination of Ethylene to a Rh(CH_2_CH_2_Ph) Intermediate To form an Off-Cycle Rh(CH_2_CH_2_Ph)(η^2^-C_2_H_4_) Intermediate

To probe the kinetic relevancy of ethylene coordination and insertion for each of the oxidant systems, the effect of ethylene pressure on the reaction rate was probed. Catalysis with **Cu**^**II**^ results in an initial first-order dependence on ethylene pressure, suggesting that ethylene coordination and insertion occur before or during the rate-limiting step (Figure 7). For **Cu**^**II**^, higher ethylene pressures result in a decrease in the rate, which eventually saturates. This could suggest that ethylene coordinates with Rh to form an off-cycle intermediate. In the discussions below, we propose that ethylene insertion is the rate-limiting step for **Cu**^**II**^ catalysis. It is possible that for **Cu**^**II**^_,_ coordination of ethylene to a phenethyl intermediate occurs, resulting in an off-cycle intermediate (Scheme 10) as has been proposed previously for **Fe**^**II**^**/O**_**2**_ catalysis^74^ in addition to similar Pt,^46^ Ru,^43^ and Ir^51^ catalysts for arene alkylation. It is also possible that ethylene coordinates with the Rh carboxylate species that activates benzene C–H bonds and its coordination inhibits benzene C–H activation. Interestingly, **Cu**^**II**^**/O**_**2**_ catalysis gives an initial first-order dependence in ethylene, which saturates at high ethylene pressures, and no inverse region is observed. Similar to our observations with HOPiv, we speculate that the coordination of ethylene to Rh could inhibit an undesired reaction with dioxygen. As found in previous work,^15^ the use of **O**_**2**_ gives a first-order dependence on ethylene, which saturates at high ethylene pressure. This is consistent with ethylene insertion occurring before or during the rate-limiting step. Additionally, previous work found an initial near first-order dependence on ethylene pressure for **Fe**^**II**^**/O**_**2**_,^74^ consistent with kinetically relevant ethylene insertion, which inverts at high ethylene pressure. The inverse dependence at high pressure was attributed to a combination of (1) ethylene coordination to a Rh–phenethyl intermediate, which inhibits β-hydride elimination, and (2) ethylene coordination to the reduced form of the Fe-based oxidant, which inhibits its reoxidation by dioxygen.^74^

### Effect of Oxidant Concentration

**Figure 8:**
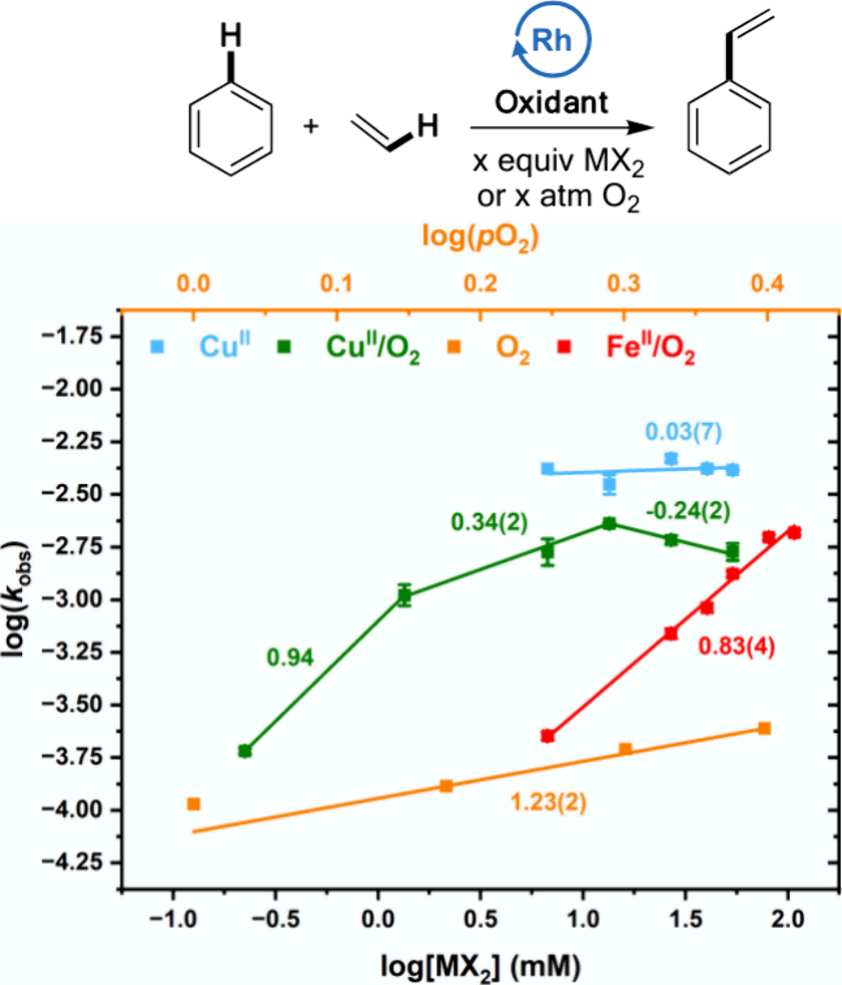
Log–log plot for the dependence of the reaction rate on direct oxidant concentration. The log[MX_2_] values represent the log of [MX_2_] in mM. Reaction conditions: **Cu**^**II**^: 7.5 mL of benzene, 70 psig of ethylene, 0.112 mM (based on single Rh atom) [(η^2^-C_2_H_4_)_2_Rh(μ-OAc)]_2_, *x* equiv of Cu(OPiv)_2_, 960 equiv of HOPiv, 120 °C. **Cu**^**II**^**/O**_**2**_: 7.5 mL benzene, 70 psig ethylene, 0.112 mM (based on single Rh atom) [(η^2^-C_2_H_4_)_2_Rh(μ-OAc)]_2_, *x* equiv Cu(OPiv)_2_, 960 equiv HOPiv, 1 atm O_2_, 150 °C. **O**_**2**_ (previously reported):^15^ 5 mL benzene, 70 psig ethylene, 0.112 mM RhCl_3_, 5 mL HOAc, *x* atm O_2_, 170 °C. **Fe**^**II**^**/O**_**2**_ (previously published):^74^ 7.5 mL of benzene, 70 psig ethylene, 0.112 mM (based on single Rh atom) [(η^2^-C_2_H_4_)_2_Rh(μ-OAc)]_2_, *x* equiv Fe(OAc)_2_, 960 equiv HOPiv, 1 atm O_2_, 150 °C. Each data point represents the average of a minimum of three independent experiments, and the error bars represent the standard deviations from the multiple experiments.

We studied the dependence of the reaction rate on the Cu(OPiv)_2_ concentration for the **Cu**^**II**^ and **Cu**^**II**^**/O**_**2**_ oxidant systems, the dioxygen partial pressure for the **O**_**2**_ system, and the Fe(OAc)_2_ concentration for the **Fe**^**II**^**/O**_**2**_ system. Following olefin insertion and β-hydride elimination, the proposed mechanisms for catalysis with each oxidant system involve the oxidation of the formed Rh–H intermediate. The results shown in Figure 8 indicate a zero-order dependence on Cu(OPiv)_2_ concentration for **Cu**^**II**^, which is consistent with previous results,^64^ suggesting that Rh–H oxidation likely occurs after the rate-limiting step. In contrast, **Cu**^**II**^**/O**_**2**_ gives an initial near first-order dependence on Cu(OPiv)_2_ concentration that saturates at higher concentrations. This finding suggests that the addition of dioxygen to an otherwise identical reaction likely results in a change in the reaction pathway or catalyst resting state. It is unlikely that the kinetics of Rh–H oxidation changes significantly upon the introduction of dioxygen to the system, unless a different active Rh species forms upon introduction of dioxygen (e.g., a Rh(III) species). As discussed above, we speculate that dioxygen reacts with active Rh(I) intermediates to form off-cycle intermediate(s). A dioxygen-coordinated species could become the catalyst resting state, which could result in Rh–H oxidation occurring before the rate-limiting step for **Cu**^**II**^**/O**_**2**_. The possible origins of differences between **Cu**^**II**^ and **Cu**^**II**^**/O**_**2**_ catalysis are discussed in more detail below. Previously published^15^ data shown in Figure 8 indicate a first-order dependence on dioxygen partial pressure for **O**_**2**_ catalysis and a near first-order dependence on Fe(OAc)_2_ concentration for **Fe**^**II**^**/O**_**2**_. These findings are consistent with Rh–H oxidation being kinetically relevant for both **O**_**2**_ and **Fe**^**II**^**/O**_**2**_.

**Figure 9:**
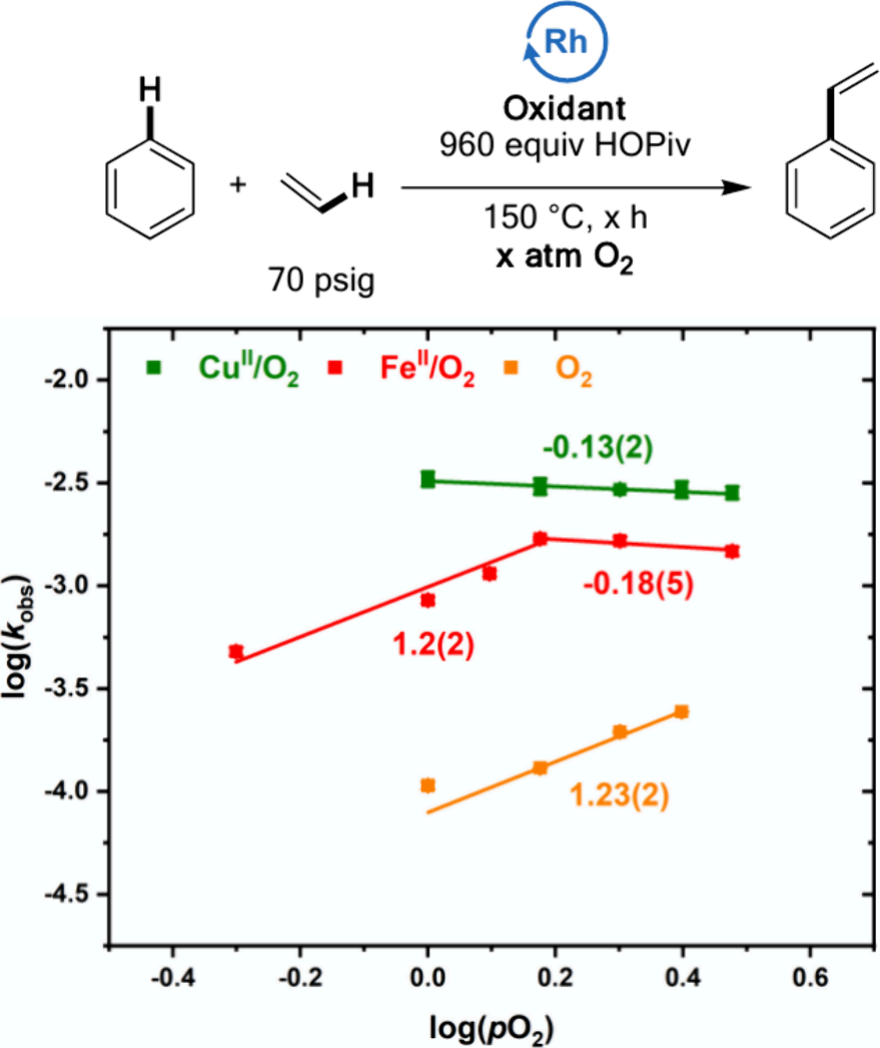
Log–log plots for the dependence of the reaction rate on dioxygen pressure when Cu(II) carboxylates or Fe(II) carboxylates are used in the presence of dioxygen. Reaction conditions: **Cu**^**II**^**/O**_**2**_: 7.5 mL of benzene, 70 psig ethylene, 0.112 mM (based on single Rh atom) [(η^2^-C_2_H_4_)_2_Rh(μ-OAc)]_2_, 480 equiv Cu(OPiv)_2_, 960 equiv HOPiv, *x* atm O_2_, 150 °C. **Fe**^**II**^**/O**_**2**_ (previously published):^74^ 7.5 mL of benzene, 70 psig ethylene, 0.112 mM (based on single Rh atom) [(η^2^-C_2_H_4_)_2_Rh(μ-OAc)]_2_, 480 equiv Fe(OAc)_2_, 960 equiv HOPiv, *x* atm O_2_, 150 °C. Each data point represents the average of a minimum of three independent experiments and the error bars represent the standard deviation from the multiple experiments.

Next, we compared the effects of the dioxygen concentration for **Fe**^**II**^**/O**_**2**_ and **Cu**^**II**^**/O**_**2**_ systems for which dioxygen reacts with a reduced form of the active oxidant to regenerate the active oxidant. As shown in Figure 9, **Cu**^**II**^**/O**_**2**_ catalysis gives a near-zero-order dependence on dioxygen concentration, and its reaction rate undergoes a statistically insignificant decrease as dioxygen pressure is increased. This suggests that reoxidation of Cu(OPiv) to Cu(OPiv)_2_ by dioxygen is not a kinetically relevant step for **Cu**^**II**^**/O**_**2**_ catalysis. In contrast, for **Fe**^**II**^**/O**_**2**_, an initial first-order dependence on dioxygen pressure is observed, which saturates at higher dioxygen pressures, suggesting that the reaction of dioxygen with the reduced form of Fe_6_(μ-OH)_2_(μ_3_-O)_2_(μ-X)_12_(HX)_2_ is a kinetically relevant step.^74^

### Effect of Rh Catalyst Concentration

Previously, we reported an order of 1.53(3) in [(η^2^-C_2_H_4_)_2_Rh(μ-OAc)]_2_ for catalysis with **Cu**^**II**^.^57^ We speculated that this is the result of an equilibrium between Cu(II)-containing mono-Rh complexes (which are less active catalysts) and bis-Rh complexes (which are more active catalysts). DFT calculations were consistent with this hypothesis, and [(η^2^-C_2_H_4_)_2_Rh(μ-OAc)]_2_(μ-Cu) as a catalyst was calculated to have a lower overall activation barrier for catalytic styrene formation than [(η^2^-C_2_H_4_)Rh(μ-OAc)_3_Cu(η^2^-C_2_H_4_).^57^ Both mono-Rh and bis-Rh Cu(II)-containing complexes were calculated to have lower activation barriers than complexes only containing Rh(I) without Cu(II).^59^

**Figure 10:**
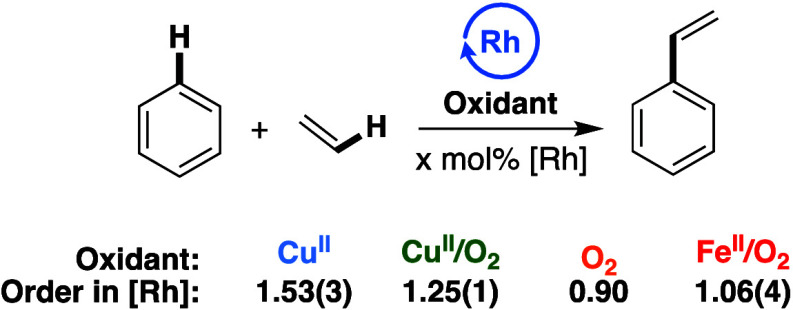
Dependence of reaction rate on Rh catalyst concentration for catalysis with each oxidant system. Reaction conditions: **Cu**^**II**^: 7.5 mL of benzene, 70 psig of ethylene, x mM (based on single Rh atom) [(η^2^-C_2_H_4_)_2_Rh(μ-OAc)]_2_, 0.480 mol % Cu(OPiv)_2_, 0.960 mol % HOPiv, 120 °C. **Cu**^**II**^**/O**_**2**_: 7.5 mL benzene, 70 psig ethylene, x mM (based on single Rh atom) [(η^2^-C_2_H_4_)_2_Rh(μ-OAc)]_2_, 0.480 mol % Cu(OPiv)_2_, 0.960 mol % HOPiv, 1 atm O_2_, 150 °C. **O**_**2**_ (previously reported)^15^ 5 mL of benzene, 70 psig ethylene, x mM RhCl_3_, 5 mL HOAc, 1 atm air, 170 °C. **Fe**^**II**^**/O**_**2**_ (previously published):^74^ 7.5 mL of benzene, 70 psig ethylene, x mM (based on single Rh atom) [(η^2^-C_2_H_4_)_2_Rh(μ-OAc)]_2_, 0.480 mol % Fe(OAc)_2_, 0.960 mol % HOPiv, 1 atm O_2_, 150 °C. Each data point represents the average of a minimum of three independent experiments, and the values in parentheses represent the standard deviations from the multiple experiments.

The order in Rh was studied as a function of the oxidant identity (Figure 10). It was found that **Cu**^**II**^**/O**_**2**_ gives an order of 1.25(1) in Rh, potentially indicating that a similar equilibrium between mono- and bis-Rh complexes to that proposed for **Cu**^**II**^ occurs.^57^ We note that the **Cu**^**II**^ reactions were carried out at 120 °C while the **Cu**^**II**^**/O**_**2**_ reactions were carried out at 150 °C, so the differences in order could be attributable to either the change in reaction conditions or the effects from dioxygen. In previous studies,^15,74^ we reported orders of 0.90 and 1.06(4), respectively, for catalysis with **O**_**2**_ and **Fe**^**II**^**/O**_**2**_. Differences in order with respect to catalyst concentration between the oxidant systems are further evidence of the possibility of distinct active catalyst species for each of the processes. The similar orders between one and two in Rh observed for **Cu**^**II**^ and **Cu**^**II**^**/O**_**2**_ catalysis could indicate that catalyst speciation (i.e., Rh(I) heteronuclear complexes with Cu^II^) is similar for the two processes.

### Linear Branched Regioselectivity

**Scheme 11:**
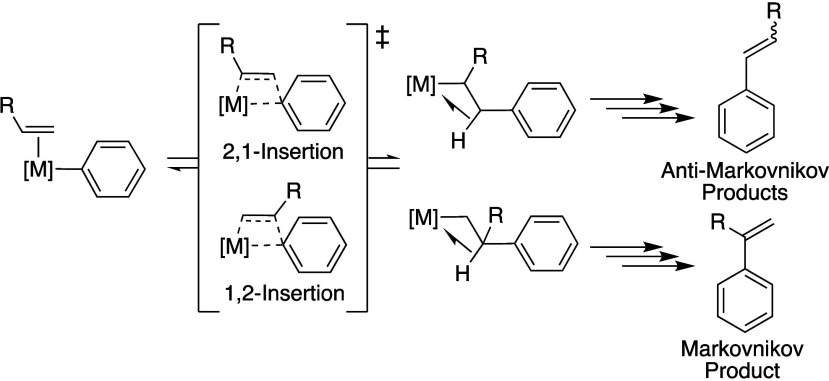
Depiction of 1,2- and 2,1-Insertion of a Mono-Substituted Olefin into a M–Ph Bond and Markovnikov and Anti-Markovnikov Products That Ultimately Form

A feature of transition metal-catalyzed arene alkenylation reactions useful for reactions with substituted olefins is that the proposed mechanisms operate through an olefin insertion step. This contrasts with acid-catalyzed mechanisms such as Friedel–Crafts arene alkylation that operate through olefin protonation and accordingly exclusively produce Markovnikov (branched) alkyl arene products for reactions with substituted olefins.^81,82^ For transition-metal-catalyzed arene alkenylation when monosubstituted olefins are used, the olefin insertion can occur either by a 1,2- or 2,1-insertion to form intermediates that can lead to the production of Markovnikov and anti-Markovnikov alkenyl arene products, respectively (Scheme 11).^15,31,58,62,63,68^ The anti-Markovnikov to Markovnikov (linear:branched) selectivity can be dependent upon factors beyond the relative kinetics of 1,2- and 2,1-insertion, including the relative stabilities of the formed alkyl aryl intermediates and the relative kinetics of the subsequent β-hydride elimination, olefin dissociation, and Rh–H oxidation steps (i.e., Curtin–Hammett conditions). These factors are dependent upon the catalyst structure and reaction pathway, both of which are influenced by the in situ oxidant.

**Figure 11:**
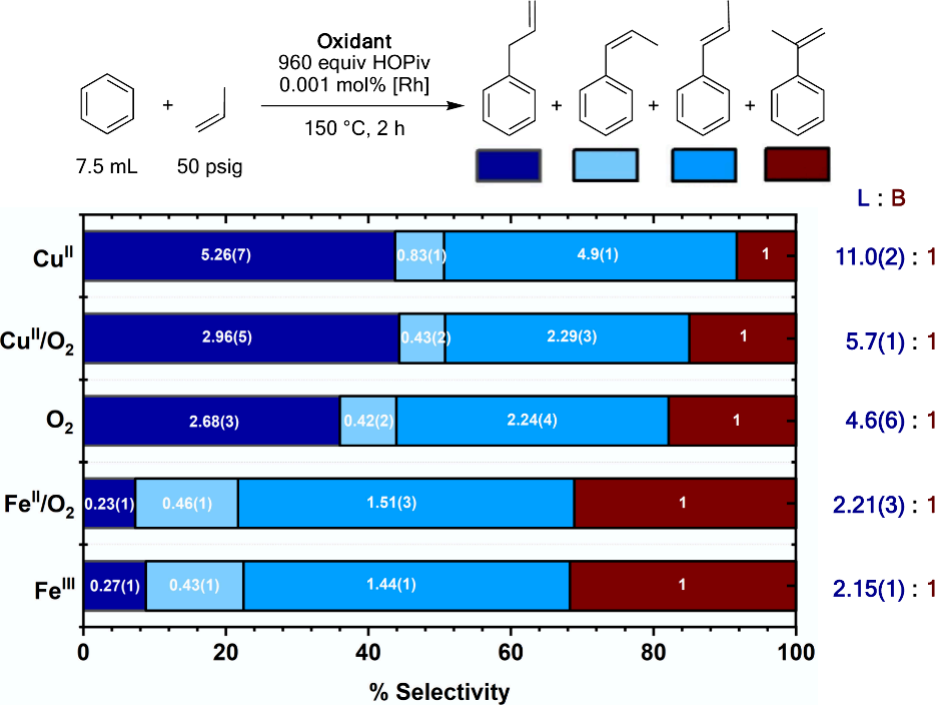
Selectivity of benzene propenylation as a function of oxidant identity. Reaction conditions: 7.5 mL of benzene, 0.001 mol % (based on single Rh atom) [(η^2^-C_2_H_4_)_2_Rh(μ-OAc)]_2_, 960 equiv (relative to single Rh atom) HOPiv, 50 psig propylene, 150 °C, 2 h, and the following for each oxidant system: **Cu**^**II**^: 480 equiv Cu(OPiv)_2_, **Cu**^**II**^**/O**_**2**_: 480 equiv of Cu(OPiv)_2_ and 1 atm dioxygen, **O**_**2**_: 1 atm dioxygen, **Fe**^**II**^**/O**_**2**_: 480 equiv Fe(OAc)_2_ and 1 atm O_2_, **Fe**^**III**^: 80 equiv Fe_6_(μ-OH)_2_(μ_3_-O)_2_(μ-OPiv)_12_(HOPiv)_2_. Each data point represents the average of a minimum of three independent experiments. Standard deviations are shown in parentheses but omitted from the graphical representation for readability.

The selectivity of propylene oxidative hydrophenylation was studied for each of the oxidant systems. As shown in Figure 11, the use of **Cu**^**II**^ gives 11.0(2):1 selectivity for anti-Markovnikov “linear” products allylbenzene, *cis*-β-methylstyrene and *trans*-β-methylstyrene over the Markovnikov “branched” product α-methylstyrene. **Cu^II^/O_2_** gives a significantly lower linear:branched ratio of 5.7(1):1. Among the linear products, allylbenzene and cis-β-methylstyrene are produced with a similar percentage of product distributions, but **Cu**^**II**^ produces ∼10% more trans-β-methylstyrene than does **Cu**^**II**^**/O**_**2**_, which accounts for the decrease in linear:branched ratio when moving from **Cu**^**II**^ to **Cu**^**II**^**/O**_**2**_. These findings are consistent with our findings throughout our studies of distinct reaction pathways or active catalysts for **Cu**^**II**^ versus **Cu**^**II**^**/O**_**2**_. As discussed above, linear:branched selectivity is dependent upon (1) differences in catalyst structure, which can influence the selectivity for 1,2- vs 2,1-olefin insertion, and (2) differences in the kinetics of Rh–H oxidation, which could be the result of a different active species and/or reaction pathway. Differences in Rh–H oxidation kinetics could result in Curtin-Hammet control over selectivity due to reversible olefin insertion.^15^ With **O**_**2**_ as the oxidant, similar selectivity between the linear products is observed to those for **Cu**^**II**^ and **Cu**^**II**^**/O**_**2**_, and there is a slight increase in α-methylstyrene production relative to **Cu^II^/O**_2_. With **Fe^II^/O_2_** and **Fe**^**III**^ systems, there is a substantial decrease in linear:branched selectivity to 2.21(3):1 and 2.15(1):1, respectively. Additionally, substantially less allylbenzene and slightly more cis-β-methylstyrene is observed for **Fe^II^/O_2_** and **Fe**^**III**^ relative to the **Cu**^**II**^, **Cu^II^/O_2_**, and **O**_**2**_ catalysis. The similarity between **Fe^II^/O_2_** and **Fe**^**III**^ supports our hypothesis of no significant change in reaction mechanism or catalyst speciation upon the addition or exclusion of dioxygen when Fe-based oxidants are used (see above).

**Figure 12:**
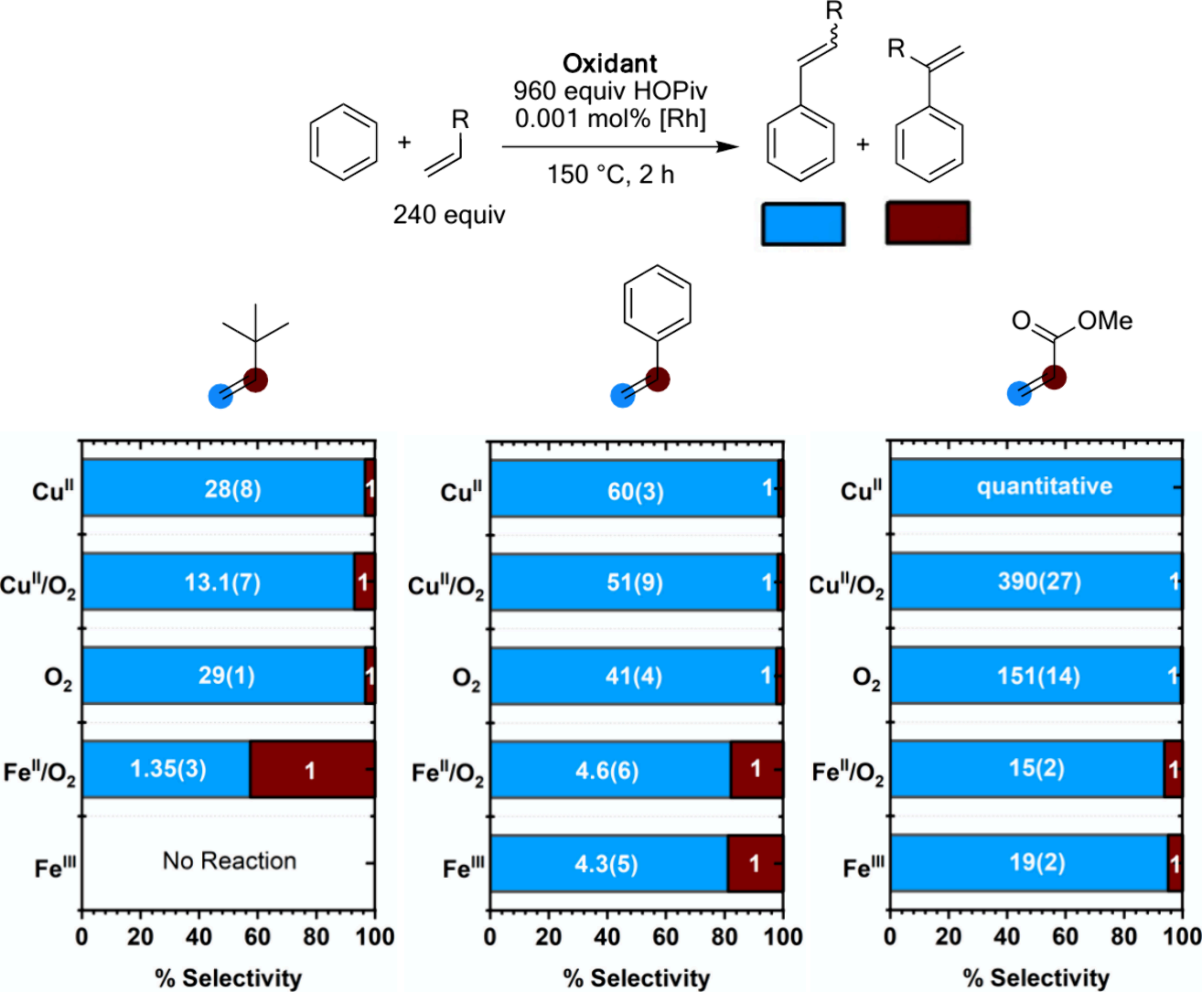
Linear:branched selectivity of benzene alkenylation with *tert-*butyl ethylene, styrene, and methyl acrylate as a function of oxidant identity. Reaction conditions: 7.5 mL of benzene, 0.001 mol % (based on single Rh atom) [(η^2^-C_2_H_4_)_2_Rh(μ-OAc)]_2_, 960 equiv (relative to single Rh atom) HOPiv, 240 equiv olefin, 150 °C, 2 h, and the following for each oxidant system: **Cu**^**II**^: 480 equiv Cu(OPiv)_2_, **Cu**^**II**^**/O**_**2**_: 480 equiv of Cu(OPiv)_2_ and 1 atm air, **O**_**2**_: 1 atm dioxygen, **Fe**^**II**^**/O**_**2**_: 480 equiv Fe(OAc)_2_ and 1 atm O_2_, **Fe**^**III**^: 80 equiv Fe_6_(μ-OH)_2_(μ_3_-O)_2_(μ-OPiv)_12_(HOPiv)_2_. Each data point represents the average of a minimum of three independent experiments. Standard deviations are shown in parentheses but have been omitted from the graphical representation for readability.

To probe the generality of linear:branched selectivity trends among the five oxidant systems shown in Figure 11, studies were extended to sterically bulky *tert-*butyl ethylene, styrene, and the Michael acceptor methyl acrylate (Figure 12). With *tert-*butyl ethylene as the olefin, **Cu**^**II**^ gives 28(8):1 selectivity for the linear product, while **Cu**^**II**^**/O**_**2**_ gives an approximately two-fold lower selectivity of 13.1(7):1, directly mirroring the results with propylene. Interestingly, **O**_**2**_ gives 29(1):1 linear:branched selectivity, which is different from the case of propylene, for which **Cu**^**II**^**/O**_**2**_ and **O**_**2**_ give similar selectivity. With **Fe**^**II**^**/O**_**2**_, 1.35(3):1 selectivity for the linear product is observed, which is lower than the linear:branched selectivity observed with propylene as the olefin. This could suggest that the electron-donating ability of the *tert-*butyl substituent plays a more important role in determining linear:branched selectivity for **Fe**^**II**^**/O**_**2**_ than sterics. With styrene as the olefin, **Cu**^**II**^ and **Cu**^**II**^**/O**_**2**_ give statistically identical linear:branched ratios of 60(3):1 and 51(9):1. **O**_**2**_ results in a slightly lower linear:branched ratio than **Cu**^**II**^ and **Cu**^**II**^**/O**_**2**_ of 41(4):1. Even with the electron-withdrawing and sterically bulky nature of the phenyl substituent, which are both expected to improve selectivity for the linear products, **Fe**^**II**^**/O**_**2**_ and **Fe**^**III**^ catalysis give only 4.6(6):1 and 4.3(5):1 linear:branched selectivity, respectively.^83,84^ With methyl acrylate as the olefin, **Cu**^**II**^ gives no detectable branched product, and only a trace branched product is observed for the **Cu**^**II**^**/O**_**2**_ and **O**_**2**_ systems. **Fe**^**II**^**/O**_**2**_ and **Fe**^**III**^ catalysis produce more substantial quantities of branched product with methyl acrylate as the olefin relative to the other oxidant systems, yielding 15(2):1 and 19(2):1 linear:branched selectivity, respectively. Using *tert*-butyl ethylene as the olefin, catalysis with **Fe**^**III**^ results in no detectable product formation, while **Fe**^**II**^**/O**_**2**_ catalysis produces a small quantity of products.

The linear:branched selectivity among the five oxidant systems probed generally follows the trend **Cu**^**II**^ > **Cu**^**II**^**/O**_**2**_ > **O**_**2**_ > **Fe**^**II**^**/O**_**2**_ ≈ **Fe**^**III**^. Similar linear:branched selectivity was generally observed between **Fe**^**II**^**/O**_**2**_ and **Fe**^**III**^, suggesting that similar active catalysts and reaction mechanisms potentially exist for these two processes. In contrast, **Cu**^**II**^ and **Cu**^**II**^**/O**_**2**_ give differences in linear:branched selectivity among the four olefin substrates probed, with **Cu**^**II**^ being more selective for linear products in all cases. The increased selectivity for linear products observed with **Cu**^**II**^ could be the result of either a unique active species versus **Cu**^**II**^**/O**_**2**_ or differences in reaction pathway relative energetics. Neither **Cu**^**II**^**/O**_**2**_ nor **Fe**^**II**^**/O**_**2**_ consistently gives linear:branched selectivity closely resembling those observed for catalysis with **O**_**2**_, providing evidence that the active species and/or reaction pathway for **O**_**2**_ catalysis is unique from the aerobic catalysis with metal carboxylate additives. We believe that this is partially attributable to Cu and Fe being imbedded in the active catalysts.^57,59,66,68^

### Studies of Ortho/Meta/Para Regioselectivity with Mono-Substituted Arenes

**Figure 13:**
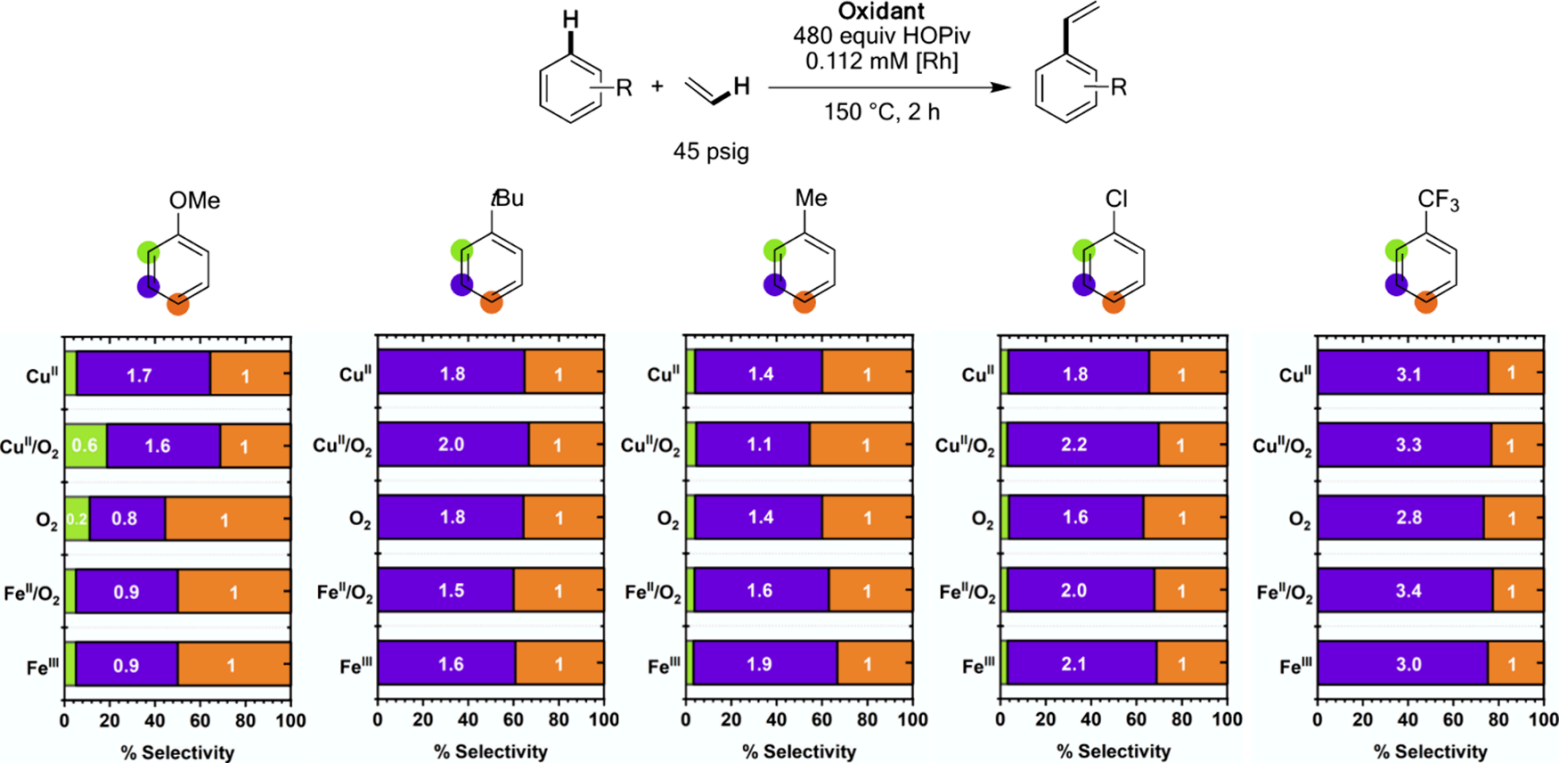
Selectivity of monosubstituted arene ethenylation as a function of oxidant identity using anisole, *tert-*butylbenzene, toluene, chlorobenzene, or α,α,α-trifluorotoluene as the arene. Reaction conditions: 2.5 or 5 mL of arene, 0.112 mM (based on a single Rh atom) [(η^2^-C_2_H_4_)_2_Rh(μ-OAc)]_2_, 480 equiv (relative to single Rh atom) HOPiv, 45 psig ethylene, 150 °C, 2 h, and the following for each oxidant system: **Cu**^**II**^**:** 240 equiv Cu(OPiv)_2_**Cu**^**II**^**/O**_**2**_: 240 equiv of Cu(OPiv)_2_ and 1 atm air (previously published),^57^ O_2_: 1 atm dioxygen, **Fe**^**II**^**/O**_**2**_: 240 equiv Fe(OAc)_2_ and 1 atm O_2_, **Fe**^**III**^: 40 equiv Fe_6_(μ-OH)_2_(μ_3_-O)_2_(μ-OPiv)_12_(HOPiv)_2_. Note: for reactions in which no ortho product was detected, it is not shown. For reactions in which the trace ortho product was observed, it was represented as 0.1-fold the quantity of the para product. Each data point represents the average of a minimum of three independent experiments. In each case, standard deviations for the ratio of meta/para products were <0.1, so the standard deviation values are not shown in the graphic.

To understand differences in arene C–H bond activation, ortho/meta/para regioselectivity was studied with anisole, *tert-*butylbenzene, toluene, chlorobenzene, and α,α,α-trifluorotoluene (Figure 13). With anisole as the substrate, **Cu**^**II**^ and **Cu**^**II**^**/O**_**2**_ give similar meta/para regioselectivities of 1.7:1 and 1.6:1, respectively. Interestingly, **Cu**^**II**^**/O**_**2**_ gives substantially more ortho product than **Cu**^**II**^. With anisole, **O**_**2**_ gives a 0.2:0.8:1 ortho/meta/para ratio, perhaps suggesting a slight electronic preference toward the ortho and para positions. **Fe**^**II**^**/O**_**2**_ and **Fe**^**III**^ catalysis gives minimal ortho product for anisole ethenylation and 0.9:1 meta/para ratios. With *tert-*butyl benzene as the arene, **Cu**^**II**^, **Cu**^**II**^**/O**_**2**_, and **O**_**2**_ give approximately 2:1 meta/para selectivity with no detectable ortho product. **Fe**^**II**^**/O**_**2**_ and **Fe**^**III**^ give slightly lower meta/para selectivities close to 1.5:1. With toluene as the arene, **Cu**^**II**^ gives a 1.4:1 meta/para ratio, and **Cu**^**II**^**/O**_**2**_ gives a 1.1:1 meta/para ratio. Toluene ethenylation using **O**_**2**_ results in a 1.4:1 meta/para ratio, and the use of **Fe**^**II**^**/O**_**2**_ and **Fe**^**III**^ gives 1.6:1 and 1.9:1 meta/para selectivity, respectively. With chlorobenzene as the arene, **Cu**^**II**^, **Cu**^**II**^**/O**_**2**_, **Fe**^**II**^**/O**_**2**_, and **Fe**^**III**^ each give approximate 2:1 meta/para regioselectivity, while **O**_**2**_ gives a lower meta/para ratio of 1.6:1. The observation of slightly lower meta/para ratios using **O**_**2**_ across substrates bearing ortho/para directing substituents could indicate that catalysis using **O**_**2**_ bears more electrophilic character in its arene C–H activation step,^57^ a possibility that was probed in more detail as described below. With α,α,α-trifluorotoluene, no ortho product was detected for catalysis with any oxidant system, and an approximate 3:1 meta/para selectivity is observed for each oxidant. Broadly, the results from our ortho/meta/para regioselectivity studies indicate (1) similar active catalysts and reaction mechanisms for **Fe**^**III**^ and **Fe**^**II**^**/O**_**2**_ catalysis, (2) distinct active catalysts and/or mechanisms for **Cu**^**II**^ and **Cu**^**II**^**/O**_**2**_ catalysis, and (3) the ortho/meta/para selectivity differences between the oxidant systems are similar to the observed linear/branched selectivity differences.

**Figure 14:**
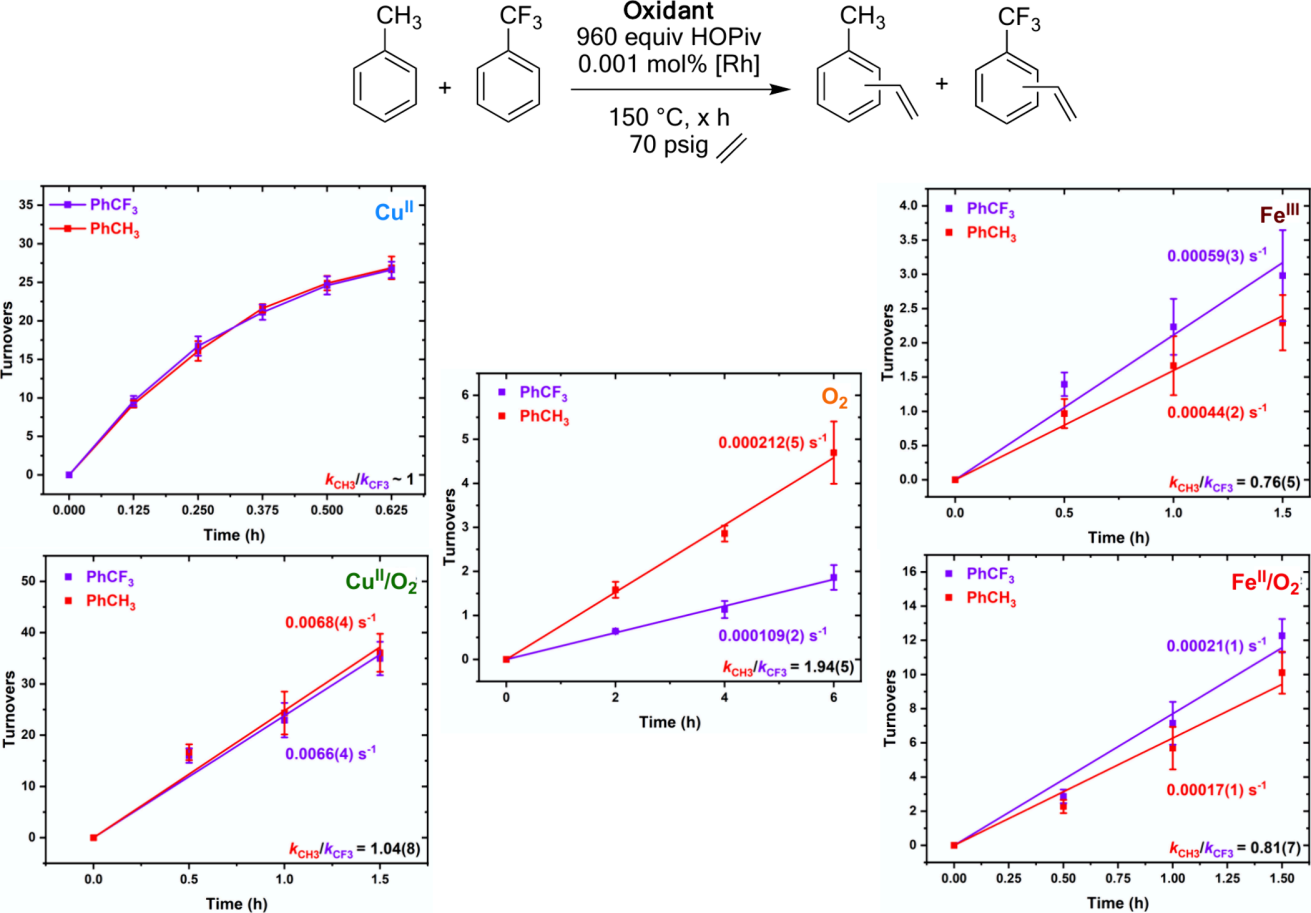
Kinetic arene ethenylation competition experiments utilizing equimolar quantities of α,α,α-trifluorotoluene and toluene. Reaction conditions: 0.0352 mol toluene, 0.0352 mol α,α,α-trifluorotoluene, 0.112 mM (based on single Rh atom) [(η^2^-C_2_H_4_)_2_Rh(μ-OAc)]_2_, 960 equiv (relative to single Rh atom) HOPiv, 50 psig propylene, 150 °C, 2 h, and the following for each oxidant system: **Cu**^**II**^: 480 equiv Cu(OPiv)_2_ (previously published),^57^**Cu**^**II**^**/O**_**2**_: 480 equiv of Cu(OPiv)_2_ and 1 atm air, **O**_**2**_: 1 atm dioxygen, **Fe**^**II**^**/O**_**2**_: 480 equiv Fe(OAc)_2_ and 1 atm O_2_, **Fe**^**III**^: 80 equiv Fe_6_(μ-OH)_2_(μ_3_-O)_2_(μ-OPiv)_12_(HOPiv)_2_. Each data point represents the average of a minimum of three independent experiments, and error bars represent the standard deviations for multiple trials.

As reported previously for comparisons between Rh and Pd catalyzed arene alkenylation,^57^ we performed intermolecular competition experiments using equimolar quantities of toluene and α,α,α-trifluorotoluene to probe for the possible metal-based electrophilicity during arene C–H activation (Figure 14). In this previous study, we observed that the rates of toluene and α,α,α-trifluorotoluene ethenylation are statistically identical for Rh catalysis, but for Pd, catalysis toluene reacts ∼10-fold more rapidly than α,α,α-trifluorotoluene.^57^ These findings were attributed to Pd catalysis operating through an arene C–H activation mechanism with a substantial electrophilic aromatic substitution character. In contrast, we proposed that the arene C–H activation step for Rh catalysis is not significantly electrophilic. In previously published results shown in Figure 14,^57^ Rh catalysis using toluene and α,α,α-trifluorotoluene gives statistically identical turnovers versus time plots for **Cu**^**II**^ catalysis. Similarly, in new results, **Cu**^**II**^**/O**_**2**_ gives no statistically significant difference between the two substrates. Also, in new results with **O**_**2**_, toluene is vinylated 1.94(5)-fold more rapidly than α,α,α-trifluorotoluene, indicating that the Rh-mediated arene C–H activation step likely has some electrophilic character. As noted above, with Pd catalysis, we observed that toluene reacts ∼10-fold more rapidly than α,α,α-trifluorotoluene,^57^ so the effect is not as significant for Rh catalysis with **O**_**2**_ as with Pd catalysis with Cu(OPiv)_2_ as the oxidant. For catalysis with **Fe**^**II**^**/O**_**2**_ and **Fe**^**III**^, toluene reacts 0.81(7) and 0.76(5)-fold the rate of α,α,α-trifluorotoluene. α,α,α-Trifluorotoluene reacts more rapidly than toluene in each of **Fe**^**II**^**/O**_**2**_ and **Fe**^**III**^. The apparent slight preference for α,α,α-trifluorotoluene over toluene when **Fe**^**II**^**/O**_**2**_ and **Fe**^**III**^ were used could indicate a preference for substrates with more acidic C–H bonds, which could indicate that the C–H activation step has deprotonation character.

The results from intermolecular competition experiments between toluene and α,α,α-trifluorotoluene could suggest that the active catalysts for **Cu**^**II**^ and **Cu**^**II**^**/O**_**2**_ are similar despite the two catalytic systems operating through distinct reaction pathways. Additionally, the active catalyst for **O**_**2**_ likely activates arene C–H bonds through an electrophilic mechanism. Also, it is possible that a Rh(II) or Rh(III) complex, such as a Rh(III) peroxo species with enhanced electrophilicity relative to Rh(I), is the active species for catalysis with **O**_**2**_.

**Scheme 12:**
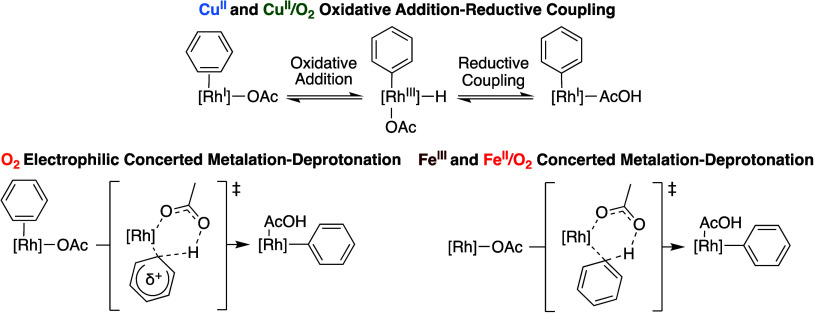
Potential Origins of Differences in Toluene versus α,α,α-Trifluorotoluene Relative Rates between the Oxidant Systems

To summarize, the results in Figure 14 indicate the following: (1) **Cu**^**II**^ and **Cu**^**II**^**/O**_**2**_ catalysis react with toluene and α,α,α-trifluorotoluene at a statistically identical rate, (2) **O**_**2**_ catalysis reacts with toluene 1.94(5)-fold more rapidly than α,α,α-trifluorotoluene, and (3) **Fe**^**III**^ and **Fe**^**II**^**/O**_**2**_ catalysis react with α,α,α-trifluorotoluene more rapidly than toluene. The absence of electronic effects for **Cu**^**II**^ and **Cu**^**II**^**/O**_**2**_ catalysis are consistent with previously reported DFT calculations, which indicate that **Cu**^**II**^ catalysis operates through a stepwise arene C–H bond oxidative addition and subsequent carboxylic acid reductive coupling,^59^ a pathway which might be insensitive to arene electronics (Scheme 12). When subjected to **O**_**2**_ catalysis, toluene reacts more rapidly than α,α,α-trifluorotoluene, possibly indicating that a concerted metalation-deprotonation mechanism with electrophilic character is active.^85^ In contrast, α,α,α-trifluorotoluene reacts slightly more rapidly than toluene when subjected to **Fe**^**III**^ and **Fe**^**II**^**/O**_**2**_ catalysis, which could indicate that a classical concerted metalation-deprotonation pathway is active.^86−88^

### Comparison of **Cu^II^** and **Cu^II^/O_2_** Catalysis

Studies of benzene ethenylation kinetics, KIEs, reagent concentration dependence, and selectivity with monosubstituted olefins and arenes suggest that distinct reaction mechanisms and/or active species exist between catalysis with **Cu**^**II**^ and **Cu**^**II**^**/O**_**2**_ oxidants. Previously, we reported that [(η^2^-C_2_H_4_)_2_Rh(μ-OAc)]_2_ reacts with Cu(OPiv)_2_ to form heterometallic complexes, which likely exist in complicated equilibria between mono- and bis-Rh complexes.^57,59^ Although the complicated reaction mechanisms and catalyst speciation make definitive conclusions about mechanistic differences between **Cu**^**II**^ and **Cu**^**II**^**/O**_**2**_ challenging, below, we speculate about a possible rationale for our observations.

As shown in Figure 1, the rate of styrene production is slower with **Cu**^**II**^**/O**_**2**_ than with **Cu**^**II**^, and apparent catalyst deactivation occurs over time for **Cu**^**II**^**/O**_**2**_. The differences in reagent concentration dependencies, KIEs, and selectivity with monosubstituted arenes and olefins between **Cu**^**II**^ and **Cu**^**II**^**/O**_**2**_ suggest that **Cu**^**II**^**/O**_**2**_ catalysis proceeds by a unique reaction pathway and/or with a different active catalyst.

A possible rationale for differences in catalysis between **Cu**^**II**^ and **Cu**^**II**^**/O**_**2**_ is the formation of a different active catalyst for **Cu**^**II**^**/O**_**2**_, as a result of Rh reacting with dioxygen. The existence of a different active species for **Cu**^**II**^**/O**_**2**_ would be consistent with the observed differences in ortho/meta/para and linear:branched regioselectivity, KIEs, and reagent concentration dependencies versus **Cu**^**II**^. The results from kinetic competition experiments using equimolar quantities of toluene and α,α,α-trifluorotoluene indicate that **Cu**^**II**^ and **Cu**^**II**^**/O**_**2**_ operate through similar arene C–H activation mechanisms. In contrast, **O**_**2**_ catalysis reacts with toluene ∼2-fold faster than α,α,α-trifluorotoluene, which suggests that an electrophilic C–H activation pathway might be active for **O**_**2**_ catalysis. Additionally, both **Cu**^**II**^ and **Cu**^**II**^**/O**_**2**_ catalysis give orders in Rh concentration between one and two, which is distinct from the **O**_**2**_ and **Fe**^**II**^**/O**_**2**_ catalysis, which give near first-order dependencies. These observations possibly indicate that the catalysts for **Cu**^**II**^ and **Cu**^**II**^**/O**_**2**_ catalysis are similar in nature.

Another possible origin of differences between **Cu**^**II**^ and **Cu**^**II**^**/O**_**2**_ catalysis is that dioxygen reacts with in-cycle Rh(I) intermediates to form off-cycle intermediates. For this proposal, the speciation of in-cycle intermediates could be similar to those for **Cu**^**II**^ catalysis, but the reaction pathway changes as a result of dioxygen undergoing a reaction with in-cycle intermediates. Dioxygen could undergo selective reaction with a particular intermediate to form an off-cycle species, which could become the catalyst resting state(s). A change in catalyst resting state(s) could result in reaction steps that occur after the rate-limiting step for **Cu**^**II**^ catalysis occurring prior to the rate-limiting step for **Cu**^**II**^**/O**_**2**_.

**Scheme 13:**
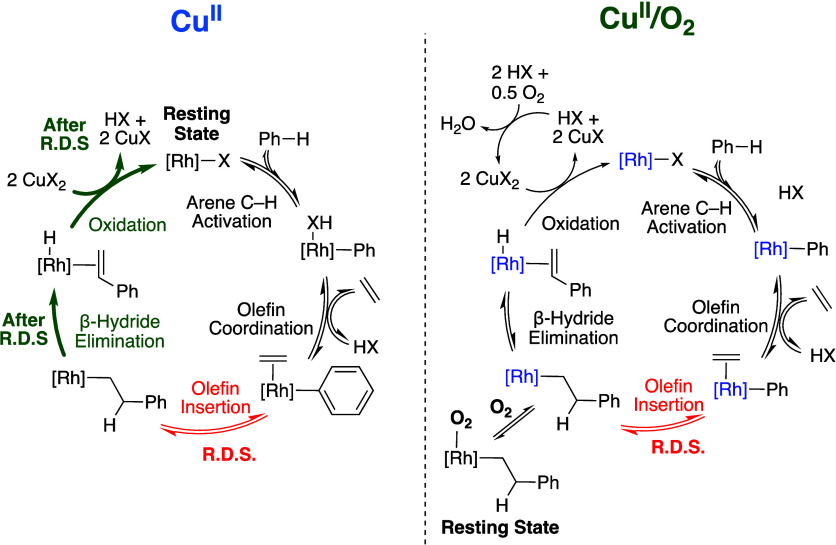
Possible Differences in Reaction Pathways between Catalysis with **Cu**^**II**^ and **Cu**^**II**^**/O**_**2**_ For **Cu**^**II**^**/O**_**2**_ catalysis, dioxygen can possibly coordinate with each intermediate to form off-cycle species, however, only the proposed dominant off-cycle intermediate is shown. The use of blue text color for **Cu**^**II**^**/O**_**2**_ represents the possibility that the active catalyst for **Cu**^**II**^**/O**_**2**_ is unique to that for **Cu**^II^.

The most significant difference between **Cu**^**II**^ and **Cu**^**II**^**/O**_**2**_ is the observation of a first-order dependence on Cu(OPiv)_2_ concentration for **Cu**^**II**^**/O**_**2**_ while **Cu**^**II**^ gives a zero-order dependence in Cu(OPiv)_2_. This result suggests that Rh–H oxidation occurs before or during the rate-limiting step for **Cu**^**II**^**/O**_**2**_ and after the rate-limiting step for **Cu**^**II**^. Results herein and previous results indicate that ethylene insertion is the probable rate-limiting step for **Cu**^**II**^ catalysis,^64^ and that the Rh carboxylate species that activate the C–H bond of benzene is the catalyst resting state. If it is assumed that **Cu**^**II**^ and **Cu**^**II**^**/O**_**2**_ have similar active catalysts, then olefin insertion would likely remain the rate-limiting step for **Cu**^**II**^**/O**_**2**_. Operating under this assumption, the resting state for **Cu**^**II**^**/O**_**2**_ could be a species formed by the reaction of either a Rh–phenethyl or Rh–H intermediate with dioxygen. It should be emphasized that dioxygen can possibly react with multiple Rh(I) intermediates in the catalytic cycle; however, its selective reaction with a Rh–H or Rh–phenethyl intermediate to form an off-cycle intermediate which serves as the catalyst resting state is most consistent with our observations. Since the Rh–phenethyl intermediate has a vacant coordination site following the insertion of ethylene into a Rh–Ph bond, we show coordination of dioxygen to a Rh–phenethyl in Scheme 13. Under this proposed mechanism, oxidation of a Rh–H intermediate and benzene C–H activation occur prior to the rate-limiting olefin insertion step. These proposals are consistent with our observation of a first-order dependence on Cu(OPiv)_2_ concentration, primary KIE when using C_6_H_6_ versus C_6_D_6_, and a first-order dependence on ethylene pressure for **Cu**^**II**^**/O**_**2**_. Our experimental studies indicate that **Cu**^**II**^ and **Cu**^**II**^**/O**_**2**_ catalytic processes are likely catalyzed by different active species and proceed through different reaction pathways (Scheme 13).

For catalysis with **O**_**2**_, our laboratory previously observed kinetically relevant arene C–H activation, ethylene insertion, and the oxidation of Rh–H by dioxygen. We speculated that Rh–H oxidation by dioxygen is likely the rate-limiting step.^15^ It is possible that the resting state for **O**_**2**_ is similar to **Cu**^**II**^**/O**_**2**_, and the slower overall rate of catalysis is consistent with previous calculations that revealed decreased activation barriers when Cu(II) is imbedded in the active catalyst.^59^ In mechanistic studies of catalysis with **Fe**^**II**^**/O**_**2**_, we observed evidence for kinetically relevant arene C–H activation, ethylene insertion, oxidation of Rh–H by Fe_6_(μ-OH)_2_(μ_3_-O)_2_(μ-X)_12_(HX)_2_ and reoxidation of the reduced form of the oxidant by dioxygen. As each step was kinetically relevant for **Fe**^**II**^**/O**_**2**_, a rate-limiting step could not be conclusively identified.

## Summary and Conclusions

In this work, the influence of oxidant identity on the reaction pathway and selectivity of Rh-catalyzed arene alkenylation was studied and quantified. Our study involved comparisons of catalysis using Cu(II) carboxylates in the presence and absence of dioxygen, dioxygen as the sole in situ oxidant, and catalysis using Fe carboxylate oxidants in the presence of dioxygen. Our conclusions include:

1. The rate of benzene ethenylation follows the trend **Cu**^**II**^ ≫ **Cu**^**II**^**/O**_**2**_ > **Fe**^**III**^ = **Fe**^**II**^**/O**_**2**_ ≫ **O**_**2**_. We attribute the fast rate with **Cu**^**II**^ relative to **Cu**^**II**^**/O**_**2**_ to the coordination of dioxygen to Rh to form an inactive off-cycle intermediate(s) including possibly the catalyst resting state. **Fe**^**III**^ and **Fe**^**II**^**/O**_**2**_ catalysis give similar turnover frequencies, indicating that the presence of dioxygen is not detrimental to the reaction rate as is the case with **Cu**^**II**^**/O**_**2**_. The kinetics of styrene production using **O**_**2**_ is substantially slower than the other systems, likely partially as a result of Rh–H oxidation by dioxygen being kinetically challenging.
2. Another factor that could impact the kinetics and selectivity of reactions is active catalyst identity: previously,^59^ it was found that [(η^2^-C_2_H_4_)_2_Rh(μ-OPiv)]_2_(μ-Cu) is a likely active catalyst for the **Cu**^**II**^ system. It is possible that [(η^2^-C_2_H_4_)_2_Rh(μ-OPiv)]_2_(μ-Cu) is also an active catalyst for **Cu**^**II**^**/O**_**2**_, and reaction with dioxygen forms an off-cycle intermediate(s). It is likely that the **Fe**^**II**^**/O**_**2**_ and **Fe**^**III**^ systems also form heterometallic complexes, which could have enhanced activity over Rh complexes without Cu or Fe atoms (e.g., **O**_**2**_ conditions). In contrast, no second metal is present for **O**_**2**_ catalysis, which could be the origin of its decreased activity.
3. The initial selectivity for styrene versus undesired side products is highest with **Cu**^**II**^. **Cu**^**II**^**/O**_**2**_ catalysis forms substantial quantities of phenyl pivalate, and benzaldehyde is observed as a side product for each of the systems except **Cu**^**II**^. **Fe**^**III**^ catalysis produces ∼5% biphenyl. The production of *trans*-stilbene is dependent upon the quantity of styrene produced. As expected, more active systems produced more *trans*-stilbene.
4. Primary KIEs with unique values for each system are observed when using benzene versus benzene-d_6_ for **Cu**^**II**^, **Cu**^**II**^**/O**_**2**_, **O**_**2**_, and **Fe**^**II**^**/O**_**2**_, indicating kinetically relevant arene C–H activation. The observation of different KIE values for each system is consistent with unique active catalysts and/or mechanisms for each of the four processes. No significant KIE is observed when using deutero- or protio carboxylic acid for any of the systems.
5. At high HOPiv loading (3840 equiv relative to Rh) and low ethylene pressure (10 psig), H/D exchange between HOPiv and C_6_D_6_ is observed for catalysis with each of the oxidants, indicating that arene C–H activation is reversible for each of the oxidant systems under these reaction conditions.
6. Complicated dependencies on HOPiv and ethylene concentration with inhibition at high concentration are observed for **Cu**^**II**^ catalysis, indicating a possible change in mechanism as reaction conditions are changed. This possibility is consistent with our previously published results, suggesting that catalyst speciation is complicated and highly condition-dependent.^57,66^ Since arene C–H activation is reversible, the inverse dependence on HOPiv likely originates from HOPiv accelerating reverse C–H activation.
7. In contrast to the case of **Cu**^**II**^, **Cu**^**II**^**/O**_**2**_ gives near zero-order dependencies on ethylene and HOPiv at high concentrations possibly because their coordination to Rh inhibits undesired reactions with dioxygen.
8. **Cu**^**II**^ shows a zero-order dependence on Cu(OPiv)_2_ concentration, while **Cu**^**II**^**/O**_**2**_ gives a first-order dependence on Cu(OPiv)_2_. We attribute this finding to a possible change in the catalyst resting state, which results in Rh–H oxidation occurring before rate-limiting olefin insertion with **Cu**^**II**^**/O**_**2**_. As reported previously,^15,74^**Fe**^**II**^**/O**_**2**_ and **O**_**2**_ give first-order dependencies on the active oxidant concentration.
9. For oxidative hydrophenylation of monosubstituted olefins, **Cu**^**II**^ gives higher anti-Markovnikov selectivity than **Cu**^**II**^**/O**_**2**_, possibly originating from the change in the active catalyst identity and/or reaction pathway. Also, **O**_**2**_ and **Fe**^**II**^**/O**_**2**_ give lower linear:branched selectivity than **Cu**^**II**^.
10. Studies of ortho/meta/para regioselectivity using monosubstituted arenes suggest similar active catalysts and mechanisms for **Fe**^**III**^ and **Fe**^**II**^**/O**_**2**_ but distinct active catalysts between **Cu**^**II**^ and **Cu**^**II**^**/O**_**2**_. These findings are consistent with mechanistic studies comparing **Cu**^**II**^ and **Cu**^**II**^**/O**_**2**_, which reveal distinct reaction mechanisms for the two processes. Catalysis using **O**_**2**_ gives distinct regioselectivity to the other systems, indicating a unique active catalyst and reaction mechanism for catalysis with **O**_**2**_.
11. Kinetic intermolecular competition experiments using equimolar quantities of toluene and α,α,α-trifluorotoluene result in statistically identical rates of product formation when **Cu**^**II**^ and **Cu**^**II**^**/O**_**2**_ are used, while with **O**_**2**_ catalysis, toluene reacts ∼2-fold more rapidly than α,α,α-trifluorotoluene. Interestingly, with **Fe**^**II**^**/O**_**2**_ and **Fe**^**III**^ catalysis, α,α,α-trifluorotoluene reacts slightly more rapidly than toluene. These findings indicate that the arene C–H activation step for **Cu**^**II**^ and **Cu**^**II**^**/O**_**2**_ catalysis might occur by a mechanism with minimal electrophilic character. In contrast, the active catalyst for **O**_**2**_ likely activates C–H bonds through a mechanism with an electrophilic character, possibly indicating an electrophilic Rh(II) or Rh(III) active species. The observation of slightly faster catalysis with α,α,α-trifluorotoluene than toluene for **Fe**^**III**^ and **Fe**^**II**^**/O**_**2**_ could indicate that C–H bond acidity determines arene reactivity. This finding might indicate that the arene C–H activation step for **Fe**^**III**^ and **Fe**^**II**^**/O**_**2**_ resembles a deprotonation.

Comparative studies have illustrated that the variation of oxidant identity for Rh-catalyzed arene alkenylation significantly affects catalyst activity, longevity, reaction pathway, and regioselectivity with monosubstituted olefins and arenes. This study has demonstrated that oxidants can influence multiple steps in Rh-catalyzed arene alkenylation, aside from the Rh–H oxidation step. In addition to its value in further optimization of Rh-catalyzed arene alkenylation, these studies could aid in the future development of transition metal-catalyzed hydrocarbon oxidative functionalization reactions.

## Experimental Section

### General Consideration

Unless otherwise noted, all synthetic procedures were performed under anaerobic conditions in a dinitrogen-filled glovebox. Glovebox purity was maintained by periodic dinitrogen purges and was monitored by an oxygen analyzer (O_2_ < 15 ppm for all reactions). Benzene was obtained from a commercial source and purified using a solvent purification system with activated alumina. [(η^2^-C_2_H_4_)_2_Rh(μ-OAc)]_2_,^89^ Cu(OPiv)_2_^90^ and Fe^III^_6_(μ-OH)_2_(μ_3_-O)_2_(μ-OPiv)_12_(HOPiv)_2_^74^ were prepared according to published procedures. All other reagents were obtained from commercial sources and used as received. Quantities of [(η^2^-C_2_H_4_)_2_Rh(μ-OAc)]_2_ of less than 1 mg were obtained by dilution in the appropriate arene solvent. CAUTION: hydrocarbon/dioxygen mixtures can be explosive. Appropriate precautionary measures should be taken to avoid sparks, reactions should be performed in stainless-steel high-pressure reactors equipped with pressure relief valves, and the vessels should have pressure ratings higher than the pressure that would be reached in the event of ignition. While heating and pressurizing the vessels, they should be kept behind a blast shield to protect the operator in the event that the pressure relief valve opens. Additionally, all of the typical precautions of using high pressure must be taken. GC-MS analysis was performed using a Shimadzu GCMS-QP-2030 Plus system with a 30 m x 0.25 mm RTX-Rxi-5 ms column with 0.25 μM film thickness. A plot of peak area versus molar ratio gave a regression line using hexamethylbenzene as the internal standard. The slope and correlation coefficient of the regression lines were 0.394 and 0.999 (styrene), 0.238 and 0.993 (vinyl pivalate) 0.232 and 0.999 (benzaldehyde), 0.740 and 0.999 (phenyl pivalate), 0.964 and 0.999 (biphenyl), 0.679 and 0.999 (*trans*-stilbene), 0.348 and 0.999 (3-trifluoromethylstyrene) and 0.357 and 0.999 (3-methylstyrene). For reactions of benzene with propylene, methyl acrylate, styrene, and *tert-*butyl ethylene, products were identified by MS and the regioselectivity was calculated by relative GC-MS peak areas. For monosubstituted arene ethenylation reactions, products were identified using authentic styrene derivatives, and the ortho/meta/para regioselectivity was calculated by relative peak areas. For monosubstituted olefin oxidative hydrophenylation, products were identified by their mass spectra, and Markovnikov/anti-Markovnikov selectivity was determined by relative peak areas.

### Procedure for Probing Benzene Ethenylation Kinetics and Selectivity

Under an atmosphere of dry dinitrogen, three 10 mL vials with stir bars were charged with benzene (7.5 mL, 84.6 mol), [(η^2^-C_2_H_4_)_2_Rh(μ-OAc)]_2_ (0.184 mg, 0.846 μmol, 0.001 mol % relative to benzene per single Rh atom), and HOPiv (82.9 mg, 0.812 mmol, 960 equiv relative to single Rh atom). For the **Cu**^**II**^ and **Cu**^**II**^**/O**_**2**_ reactions, Cu(OPiv)_2_ (107.0 mg, 0.4038 mmol, 480 equiv) was added to each of the three vials, for the **Fe**^**II**^**/O**_**2**_ system, Fe(OAc)_2_ (69.4 mg, 0.404 mmol, 480 equiv) was added to each of the three vials, and for the **Fe**^**III**^ system, Fe^III^_6_(μ_4_-O_2_)(μ_3_-O)_2_(μ-OPiv)_12_(HOPiv)_2_ (122.2 mg, 0.068 mmol, 80 equiv) was added to each of the three vials. The filled vials were used as inserts in three previously described stainless-steel high-pressure reactors equipped with pressure relief valves. The reactors were sealed and subsequently pressurized with 70 psig of ethylene. For the **Cu/O**_**2**_, **O**_**2**_, and **Fe/O**_**2**_ catalysis, 1 atm of dioxygen was subsequently added to each reactor. The reactors were heated at 150 °C in an aluminum block on a hot plate and cooled to room temperature at each time point by placing the hot reactors in a room-temperature aluminum block. Upon cooling, the reactors were opened and sampled using a long needle, either in the air for **Cu**^**II**^**/O**_**2**_, **O**_**2**_, and **Fe**^**II**^**/O**_**2**_ or under a flow of dinitrogen for **Cu**^**II**^ and **Fe**^**III**^ catalysis. Next, 50 μL aliquots of the reaction solutions were measured with a micro syringe, diluted in 0.5 mL of benzene, and combined with 50 μL of an 11.1 mM solution of hexamethylbenzene in benzene to give 100 equiv of external standard hexamethylbenzene relative to single Rh atom. The benzene solutions were washed with a saturated aqueous solution of NaOH, and the organic phases were analyzed by GC-MS.

### Procedure for Mechanistic Studies with **Cu^II^** under Anaerobic Conditions

Under an atmosphere of dry dinitrogen, three 10 mL vials with stir bars were charged with benzene (7.5 mL, 84.6 mol), [(η^2^-C_2_H_4_)_2_Rh(μ-OAc)]_2_ (0.184 mg, 0.846 μmol, 0.001 mol % relative to benzene per single Rh atom), HOPiv (varying quantity or 82.9 mg, 0.812 mmol, 960 equiv relative to single Rh atom) Cu(OPiv)_2_ (varying quantity or 107.0 mg, 0.4038 mmol, 480 equiv). The filled vials were used as inserts in three previously described stainless-steel high-pressure reactors equipped with pressure relief valves. The reactors were sealed and subsequently pressurized with 70 psig or varying pressures of ethylene. The reactors were heated in an aluminum block on a hot plate at 120 °C and cooled to room temperature at each time point by placing the hot reactors in a room-temperature aluminum block. Upon cooling, the reactors were opened and sampled under a flow of dinitrogen. Next, 50 μL aliquots of the reaction solutions were measured with a micro syringe, diluted in 0.5 mL of benzene, and combined with 50 μL of an 11.1 mM solution of hexamethylbenzene in benzene to give 100 equiv of external standard hexamethylbenzene relative to single Rh atom. The benzene solutions were washed with a saturated aqueous solution of NaOH, and the organic phases were analyzed by GC-MS.

### Representative Procedure for Mechanistic Studies with **Cu^II^** under Aerobic Conditions

Under an atmosphere of dry dinitrogen, three 10 mL vials with stir bars were charged with benzene (7.5 mL, 84.6 mol), [(η^2^-C_2_H_4_)_2_Rh(μ-OAc)]_2_ (0.184 mg, 0.846 μmol, 0.001 mol % relative to benzene per single Rh atom), HOPiv (varying quantity or 82.9 mg, 0.812 mmol, 960 equiv relative to single Rh atom) Cu(OPiv)_2_ (varying quantity or 107.0 mg, 0.4038 mmol, 480 equiv). The filled vials were used as inserts in three previously described stainless-steel high-pressure reactors equipped with pressure relief valves. The reactors were sealed and subsequently pressurized with 70 psig or varying pressures of ethylene and 1 atm or varying pressures of dioxygen. The reactors were heated in an aluminum block on a hot plate at 150 °C and cooled to room temperature at each time point by placing hot reactors in a room temperature aluminum block. Upon cooling, the reactors were opened and sampled in the air. Next, 50 μL aliquots of the reaction solutions were measured with a micro syringe, diluted in 0.5 mL of benzene, and combined with 50 μL of 11.1 mM solution of hexamethylbenzene in benzene to give 100 equiv of external standard hexamethylbenzene relative to a single Rh atom. The benzene solutions were washed with a saturated aqueous solution of NaOH, and the organic phases were analyzed by GC–MS.

### Procedure for C_6_D_6_/HOPiv H/D Exchange Reactions

Under an atmosphere of dry dinitrogen, 2.5 mL of benzene-*d*_*6*_ was combined with [(η^2^-C_2_H_4_)_2_Rh(μ-OAc)]_2_ (0.061 mg, 0.280 μmol, 0.001 mol % per single Rh atom) HOPiv (109.9 mg, 1.077 mmol, 3840 equiv relative to single Rh). For the **Cu**^**II**^ and **Cu**^**II**^**/O**_**2**_ catalysis, Cu(OPiv)_2_ (35.7 mg, 0.134 mmol, 480 equiv) was added, for **Fe**^**II**^**/O**_**2**_, Fe(OAc)_2_ (23.1 mg, 0.135 mmol, 480 equiv) was added. The reaction mixtures were added to custom stainless-steel reactors equipped with pressure relief valves and pressurized with 10 psig of ethylene and 1 atm dioxygen (if present), and the total pressure was brought to 100 psig by pressurizing with dinitrogen. Prior to beginning the reactions, a 5 μL aliquot of the benzene-*d*_*6*_ solutions was taken from each reactor, diluted in 1 mL of methylene chloride, and analyzed by GC-MS. The reactors were heated on an aluminum block on a hot plate and cooled to room temperature at each time point by placing the hot reactors in a room-temperature aluminum block. A long needle was used to sample the reactors either in the air (for **Cu**^**II**^**/O**_**2**_, **O**_**2**_, and **Fe**^**II**^**/O**_**2**_ catalysis) or under a flow of dinitrogen (for **Cu**^**II**^ catalysis). At each time point, 5 μL aliquots from each reactor were combined with 1 mL of methylene chloride and analyzed by GC-MS to analyze the benzene proton incorporation. Next, to quantify styrene production, 50 μL aliquots of the reaction solutions were measured with a micro syringe, diluted in 0.5 mL of benzene, and combined with 50 μL of an 11.1 mM solution of hexamethylbenzene in benzene to give 100 equiv of external standard hexamethylbenzene relative to a single Rh atom. The benzene solutions were washed with a saturated aqueous solution of NaOH, and the organic phases were analyzed by GC–MS.

### Procedure for Monosubstituted Arene Regioselectivity Studies

Under an atmosphere of dry dinitrogen, 2.5 mL of anisole, *tert-*butylbenzene, toluene, chlorobenzene or α,α,α-trifluorotoluene was combined with [(η^2^-C_2_H_4_)_2_Rh(μ-OAc)]_2_ (0.061 mg, 0.280 μmol, 0.11 mM per single Rh atom) and HOPiv (13.7 mg, 0.134 mmol, 480 equiv relative to single Rh). For the **Cu**^**II**^ catalysis, Cu(OPiv)_2_ (17.8 mg, 0.0674 mmol, 240 equiv) was added, for **Fe**^**II**^**/O**_**2**_, Fe(OAc)_2_ (11.6 mg, 0.0674 mmol, 240 equiv) was added, for **Fe**^**III**^ catalysis, Fe^III^_6_(μ_4_-O_2_)(μ_3_-O)_2_(μ-OPiv)_12_(HOPiv)_2_ (20.4 mg, 0.0112 mmol, 40 equiv) was added. The reaction mixtures (three trials per arene per oxidant system) were added to separate cells of an Asynt multicell reactor equipped with pressure relief valves, which were subsequently sealed, and each cell was pressurized with 45 psig of ethylene. For **O**_**2**_ and **Fe**^**II**^**/O**_**2**_ reactions, the reactions were subsequently pressurized with 1 atm of dioxygen. The multi-cell reactor was placed on a hot plate and heated at 150 °C for 2 h. The reactor was removed from the hot plate and cooled to room temperature, and each cell was sampled in air. Next, 50 μL aliquots of the reaction solutions were measured with a micro syringe, diluted in 0.5 mL benzene, and combined with 50 μL of an 11.1 mM solution of hexamethylbenzene in benzene to give 100 equiv of external standard hexamethylbenzene relative to single Rh atom. The benzene solutions were washed with a saturated aqueous solution of NaOH, and the organic phases were analyzed by GC-MS. Distributions of regio-isomers were determined by relative peak areas, and products were identified using authentic monosubstituted styrene derivatives.

### Procedure for Monosubstituted Olefin Regioselectivity Studies

Under an atmosphere of dry dinitrogen, 2.5 mL of benzene was combined with [(η^2^-C_2_H_4_)_2_Rh(μ-OAc)]_2_ (0.061 mg, 0.280 μmol, 0.11 mM per single Rh atom) HOPiv (27.4 mg, 0.274 mmol, 960 equiv relative to single Rh), and 240 equiv (67.3 mmol) of styrene, methyl acrylate, or *tert-*butyl ethylene. For **Cu**^**II**^ catalysis, Cu(OPiv)_2_ (17.8 mg, 0.0674 mmol, 240 equiv) was added, for **Fe**^**II**^**/O**_**2**_, Fe(OAc)_2_ (11.6 mg, 0.0674 mmol, 240 equiv) was added, and for **Fe**^**III**^ catalysis, Fe^III^_6_(μ_4_-O_2_)(μ_3_-O)_2_(μ-OPiv)_12_(HOPiv)_2_ (20.4 mg, 0.0112 mmol, 40 equiv) was added. The reaction mixtures (three trials per arene per oxidant system) were added to either custom stainless-steel reactors (for propylene) and pressurized with 50 psig of propylene, or separate cells of an Asynt multicell reactor equipped with pressure relief valves (for reactions with methyl acrylate, styrene, and *tert-*butyl ethylene). The multicell reactor was subsequently sealed, and each cell was pressurized with 70 psig of dinitrogen. For the **O**_**2**_ and **Fe**^**II**^**/O**_**2**_ reactions, the reactions were subsequently pressurized with 1 atm of dioxygen. The multi-cell reactor or custom stainless-steel reactors were placed on a hot plate and heated at 150 °C for 2 h. The reactors were removed from the hot plate and cooled to room temperature, and each reaction mixture was sampled in air. Next, 50 μL aliquots of the reaction solutions were measured with a micro syringe, diluted in 0.5 mL of benzene, and combined with 50 μL of an 11.1 mM solution of hexamethylbenzene in benzene to give 100 equiv of external standard hexamethylbenzene relative to single Rh atom. The benzene solutions were washed with a saturated aqueous solution of NaOH, and the organic phases were analyzed by GC-MS. Distributions of regio-isomers were determined by relative peak areas, and the products were identified using the mass spectra.

## Supporting Information Available

Additional experimental details including detailed experimental procedures and data used to generate kinetic plots (PDF)
