# Supplementary material for: Rhodium-Catalyzed Arene Alkenylation: Selectivity and Reaction Mechanism as a Function of In Situ Oxidant Identity
Source: Organometallics. 2024 Sep 12;43(18):2113–31. doi: 10.1021/acs.organomet.4c00327 (PMC11423412; doi:10.1021/acs.organomet.4c00327)
Supplement: Supplementary file 1 — om4c00327_si_001.pdf [file om4c00327_si_001.pdf]

Supporting Information

Rhodium Catalyzed Arene Alkenylation: Selectivity and Reaction Mechanism as a Function of in situ Oxidant Identity

Marc T. Bennett,[†] Kwanwoo A. Park,[†] and T. Brent Gunnoe[†]*

[†]Department of Chemistry, University of Virginia, Charlottesville, Virginia, United States 22904

*Correspondence to: tbg7h@virginia.edu

Table of Contents

GC-MS calibration curves for vinyl pivalate, styrene, benzaldehyde, phenyl pivalate, biphenyl, *trans*-stilbene, 1,1-diphenylethylene, cis-stilbene, 3-methylstyrene and 3-trifluoromethylstyrene……………………..…..…………….S3

Mass spectra from GC-MS analysis for the linear and branched isomers formed by oxidative hydrophenylation of methyl acrylate………………………………………………………………………..………………………S4

Mass spectra for the linear and branched isomers formed by the oxidative hydrophenylation of *tert*-butylethylene.....S4

Representative GC-MS chromatogram for a benzene ethenylation reaction using $Cu(OPiv)_2$ as the oxidant under aerobic conditions…………………………………………………………………………………………….S5

Log-log plot for the dependence of reaction rate on the concentration of Rh for benzene ethenylation reactions using $Cu(OPiv)_2$ as the oxidant under aerobic conditions……………………………………………………….…….S5

Turnovers versus time plot for benzene or benzene-d_6 ethenylation reactions using $Cu(OPiv)_2$ as the oxidant at anaerobic conditions……………………………………………………………………………………..S6

Turnovers versus time plot for benzene or benzene-d_6 ethenylation reactions using $Cu(OPiv)_2$ as the oxidant at aerobic conditions……………………………………………………………………………………………..S7

Turnovers versus time plot for benzene ethenylation reactions at varying ethylene pressure using $Cu(OPiv)_2$ as the oxidant at anaerobic conditions………………………………………………………………………………….S7

Turnovers versus time plot for benzene ethenylation reactions at varying ethylene pressure using $Cu(OPiv)_2$ as the oxidant at aerobic conditions…………………………………………………………………...…………..S8

Turnovers versus time plot for benzene ethenylation reactions at varying HOPiv concentration using $Cu(OPiv)_2$ as the oxidant at anaerobic conditions…………………………………………………………………...………..S8

Turnovers versus time plot for benzene ethenylation reactions at varying HOPiv concentration using $Cu(OPiv)_2$ as the oxidant at aerobic conditions………………………………………………………………………………….S9

Turnovers versus time plot for benzene ethenylation reactions in the presence of either HOPiv or DOPiv using $Cu(OPiv)_2$ as the oxidant at anaerobic conditions……………………………………………………………..S10

Turnovers versus time plot for benzene ethenylation reactions in the presence of either HOPiv or DOPiv using $Cu(OPiv)_2$ as the oxidant at aerobic conditions……………………………………………………………….S10

Turnovers versus time plot for benzene ethenylation reactions at varying $Cu(OPiv)_2$ concentration using $Cu(OPiv)_2$ as the oxidant at anaerobic conditions……………………………………………………….…………………S11

Turnovers versus time plot for benzene ethenylation reactions at varying $Cu(OPiv)_2$ concentration using $Cu(OPiv)_2$ as the oxidant at aerobic conditions……………………………………………………..………………………S11

Turnovers versus time plot for benzene ethenylation reactions at varying $Fe_6(\mu\text{-}OH)_2(\mu_3\text{-}O)_2(\mu\text{-}OPiv)_{12}(\mu\text{-}HOPiv)_2$ loading………………...………………………………………………………………………………….S12

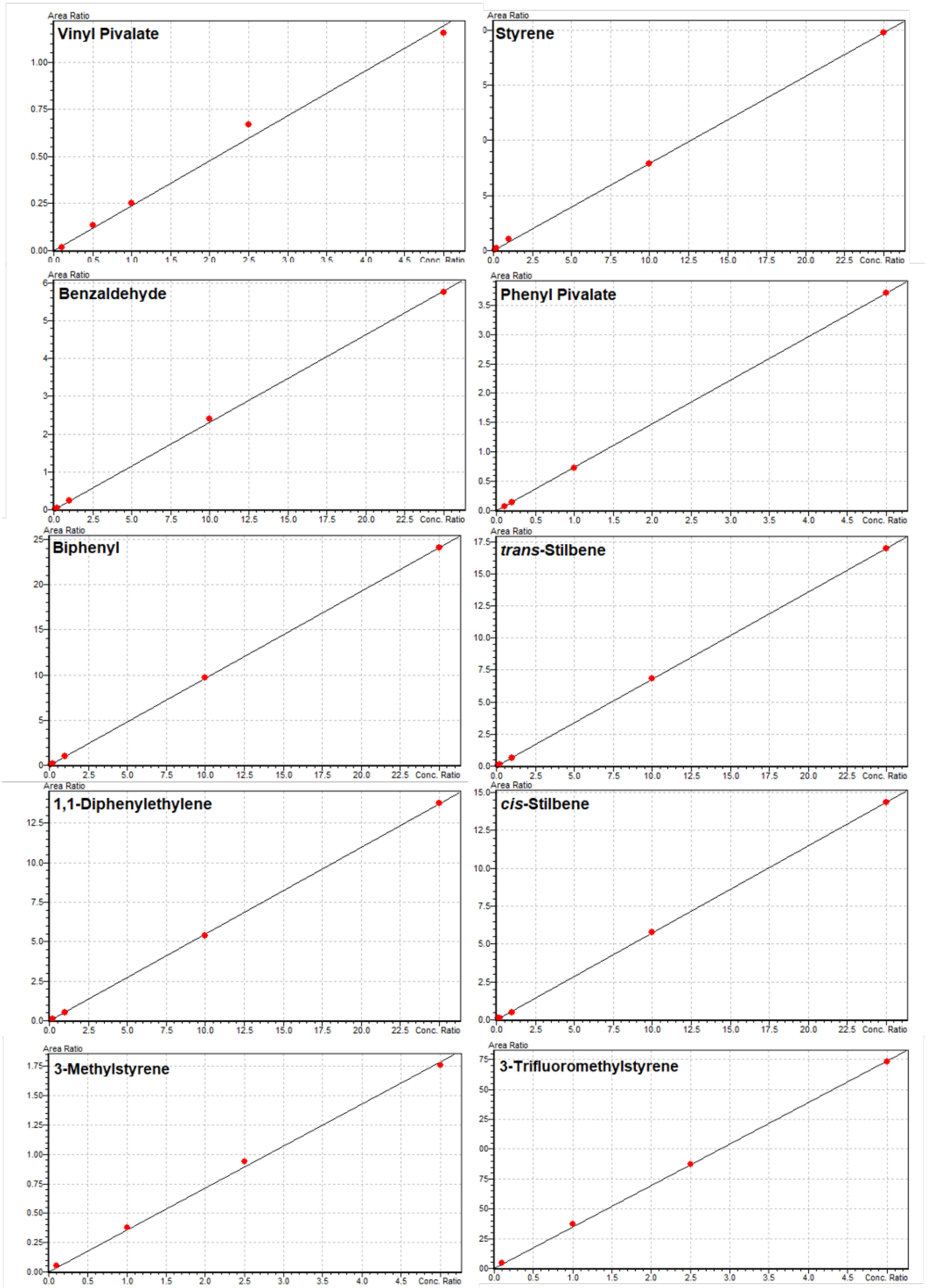

Figure S1. GC-MS calibration curves for vinyl pivalate, styrene, benzaldehyde, phenyl pivalate, biphenyl, *trans*-stilbene, 1,1-diphenylethylene, *cis*-stilbene, 3-methylstyrene and 3-trifluoromethylstyrene.

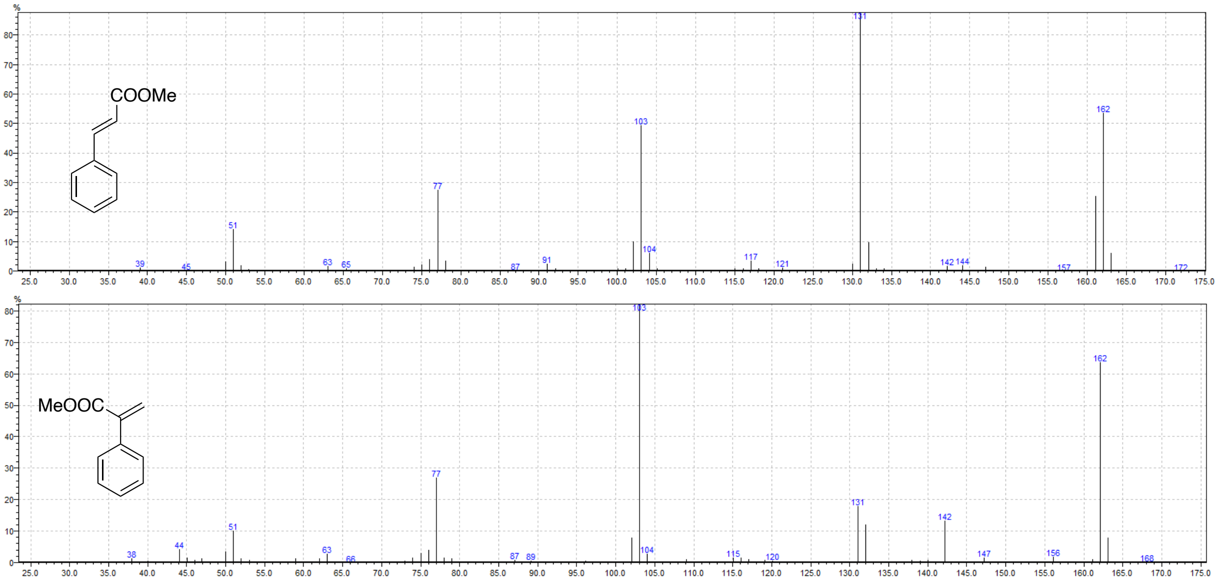

Figure S2. Mass spectra from GC-MS analysis for the linear and branched isomers formed by oxidative hydrophenylation of methyl acrylate.

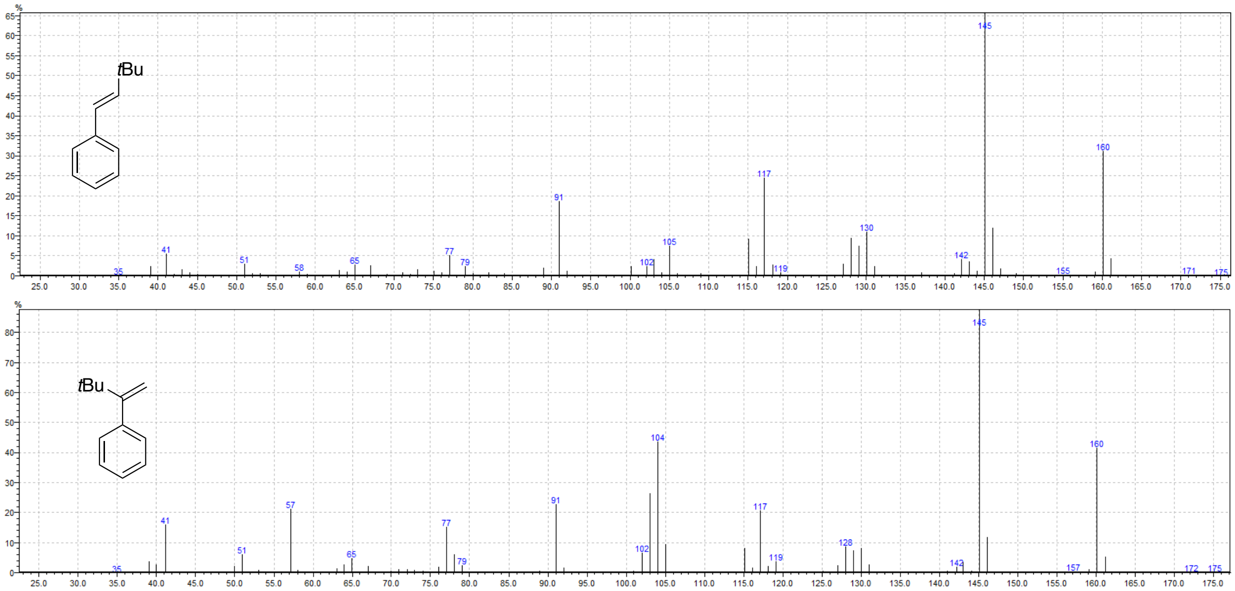

Figure S3. Mass spectra for the linear and branched isomers formed by the oxidative hydrophenylation of *tert*-butylethylene.

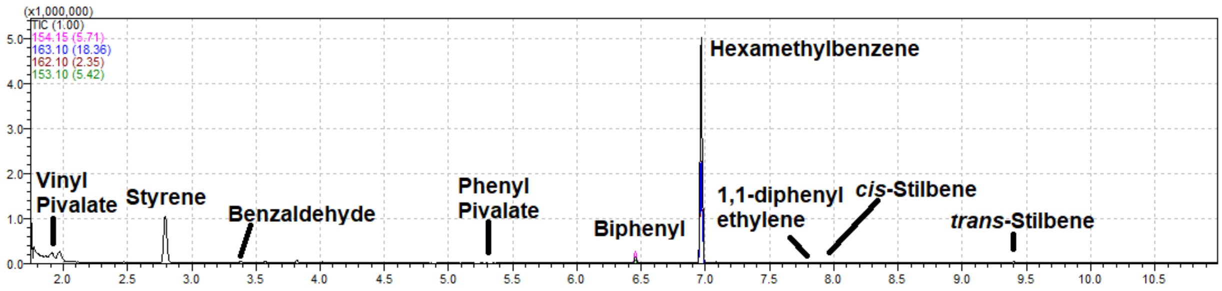

Figure S4. Representative GC-MS chromatogram for a benzene ethenylation reaction using $Cu(OPiv)_2$ as the oxidant under aerobic conditions.

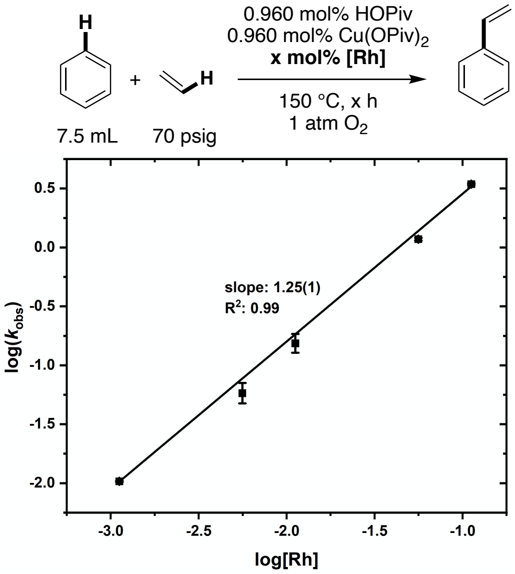

Figure S5. Log-log plot for the dependence of reaction rate on the concentration of Rh for benzene ethenylation reactions using $Cu(OPiv)_2$ as the oxidant under aerobic conditions. 7.5 mL benzene, x mol% (based on single Rh atom) $[(\eta^2\text{-}C_2H_4)_2Rh(\mu\text{-}OAc)]_2$, 0.480 mol% $Cu(OPiv)_2$, 0.960 mol% HOPiv, 70 psig ethylene, 1 atm dioxygen, 150 °C. Each data point represents the average from a minimum of three independent experiments and the error bars represent the standard deviation from the multiple experiments.

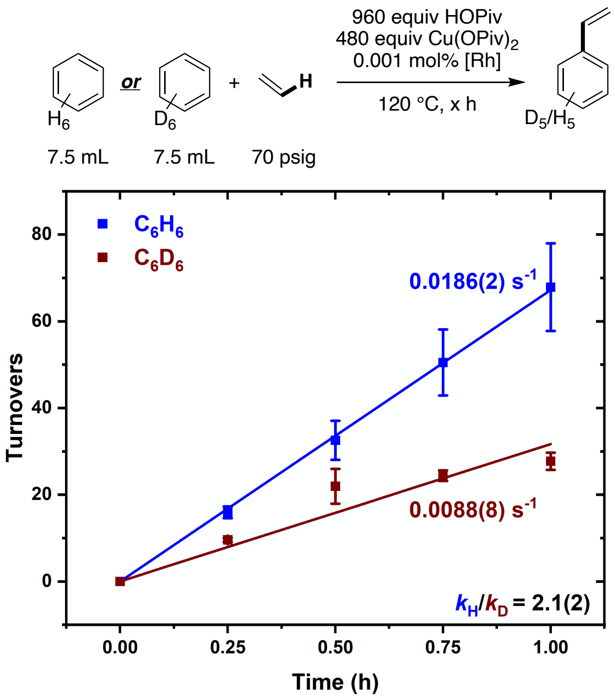

Figure S6. Turnovers versus time plot for benzene and benzene-<u>*d₆*</u> ethenylation reactions using $Cu(OPiv)_2$ as the oxidant at anaerobic conditions. Reaction conditions: 7.5 mL benzene or benzene-d_6, 0.001 mol% (based on single Rh atom) $[(\eta^2\text{-}C_2H_4)_2Rh(\mu\text{-}OAc)]_2$, 480 equiv (based on single Rh atom) $Cu(OPiv)_2$, 960 equiv HOPiv, 70 psig ethylene, 120 °C. Each data point represents the average from a minimum of three independent experiments and the error bars represent the standard deviation from the multiple experiments.

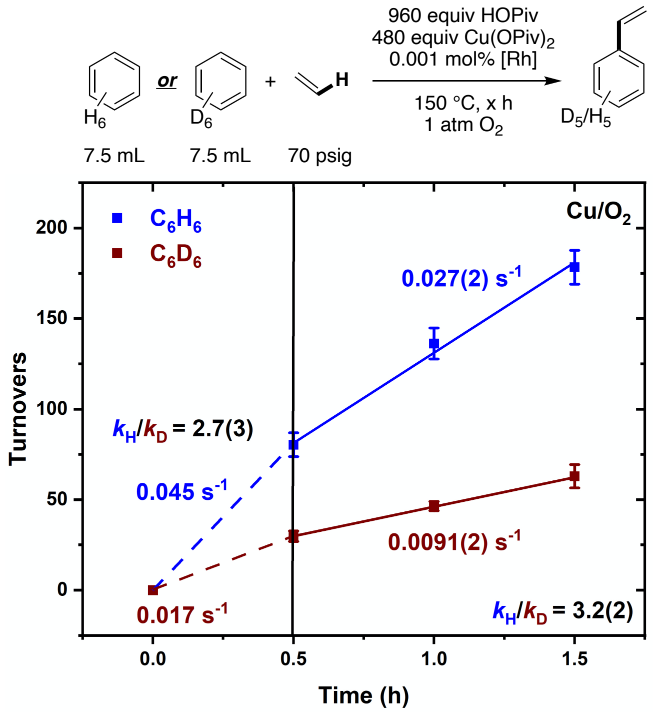

Figure S7. Turnovers versus time plot for benzene or benzene-d6 ethenylation reactions using $Cu(OPiv)_2$ as the oxidant at aerobic conditions. Reaction conditions: 7.5 mL benzene or benzene-*d6*, 0.001 mol% (based on single Rh atom) $[(\eta^2\text{-}C_2H_4)_2Rh(\mu\text{-}OAc)]_2$, 480 equiv (based on single Rh atom) $Cu(OPiv)_2$, 960 equiv HOPiv, 70 psig ethylene, 1 atm dioxygen, 150 °C. Each data point represents the average from a minimum of three independent experiments and the error bars represent the standard deviation from the multiple experiments.

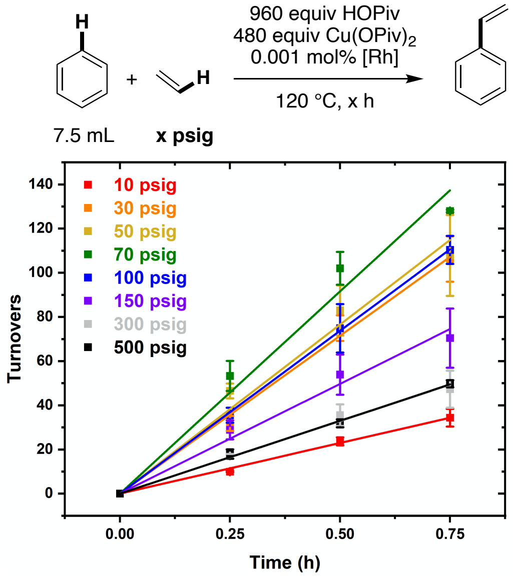

Figure S8. Turnovers versus time plot for benzene ethenylation reactions at varying ethylene pressure using $Cu(OPiv)_2$ as the oxidant at anaerobic conditions. Reaction conditions: 7.5 mL benzene, 0.001 mol% (based on single Rh atom) $[(\eta^2\text{-}C_2H_4)_2Rh(\mu\text{-}OAc)]_2$, 480 equiv (based on single Rh atom) $Cu(OPiv)_2$, 960 equiv HOPiv, 70 psig ethylene, 120 °C.

Each data point represents the average from a minimum of three independent experiments and the error bars represent the standard deviation from the multiple experiments.

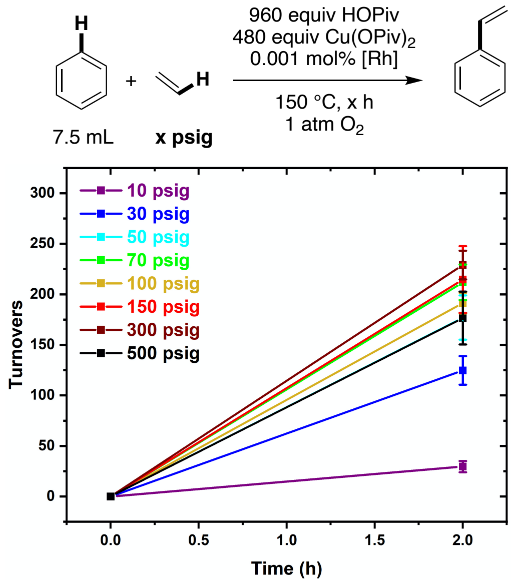

Figure S9. Turnovers versus time plot for benzene ethenylation reactions at varying ethylene pressure using Cu(OPiv)$_2$ as the oxidant at aerobic conditions. Reaction conditions: 7.5 mL benzene, 0.001 mol% (based on single Rh atom) [(η^2-C_2H_4)$_2$Rh(μ-OAc)]$_2$, 480 equiv (based on single Rh atom) Cu(OPiv)$_2$, 960 equiv HOPiv, 70 psig ethylene, 1 atm dioxygen, 150 °C. Each data point represents the average from a minimum of three independent experiments and the error bars represent the standard deviation from the multiple experiments.

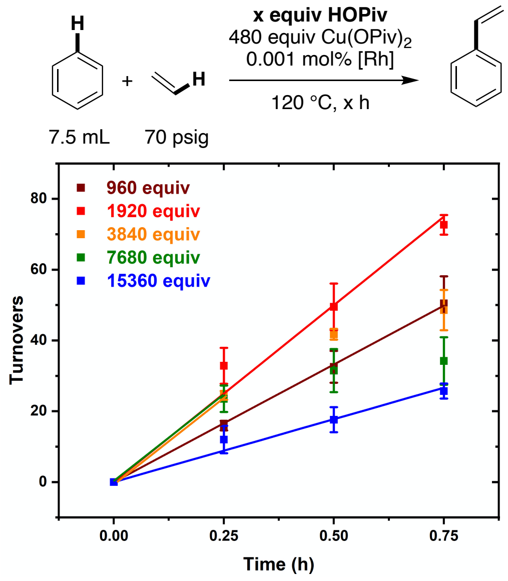

Figure S10. Turnovers versus time plot for benzene ethenylation reactions at varying HOPiv concentration using Cu(OPiv)$_2$ as the oxidant at anaerobic conditions. Reaction conditions: 7.5 mL benzene, 0.001 mol% (based on single

Rh atom) [(η^2-C_2H_4)$_2$Rh(μ-OAc)]$_2$, 480 equiv (based on single Rh atom) Cu(OPiv)$_2$, 960 equiv HOPiv, 70 psig ethylene, 120 °C. Each data point represents the average from a minimum of three independent experiments and the error bars represent the standard deviation from the multiple experiments.

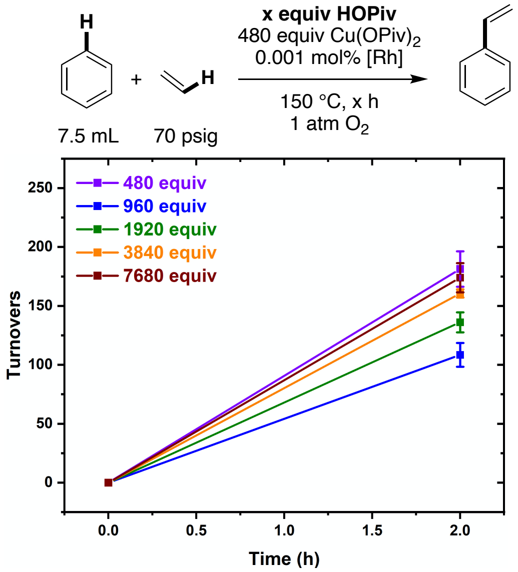

Figure S11. Turnovers versus time plot for benzene ethenylation reactions at varying HOPiv concentration using Cu(OPiv)$_2$ as the oxidant at aerobic conditions. Reaction conditions: 7.5 mL benzene, 0.001 mol% (based on single Rh atom) [(η^2-C_2H_4)$_2$Rh(μ-OAc)]$_2$, 480 equiv (based on single Rh atom) Cu(OPiv)$_2$, 960 equiv HOPiv, 70 psig ethylene, 1 atm dioxygen, 150 °C. Each data point represents the average from a minimum of three independent experiments and the error bars represent the standard deviation from the multiple experiments.

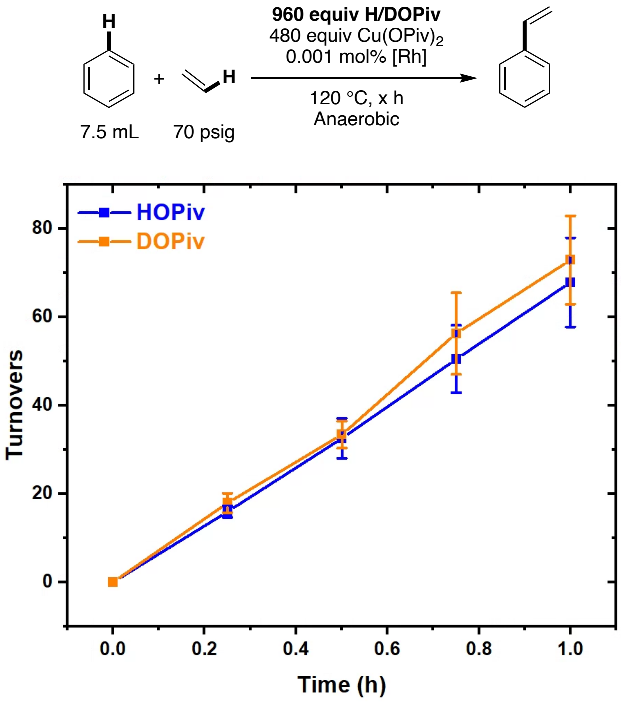

Figure S12. Turnovers versus time plot for benzene ethenylation reactions in the presence of either HOPiv or DOPiv using $Cu(OPiv)_2$ as the oxidant at anaerobic conditions. Reaction conditions: 7.5 mL benzene, 0.001 mol% (based on single Rh atom) $[(\eta^2\text{-}C_2H_4)_2Rh(\mu\text{-}OAc)]_2$, 480 equiv (based on single Rh atom) $Cu(OPiv)_2$, 960 equiv HOPiv or DOPiv, 70 psig ethylene, 120 °C. Each data point represents the average from a minimum of three independent experiments and the error bars represent the standard deviation from the multiple experiments.

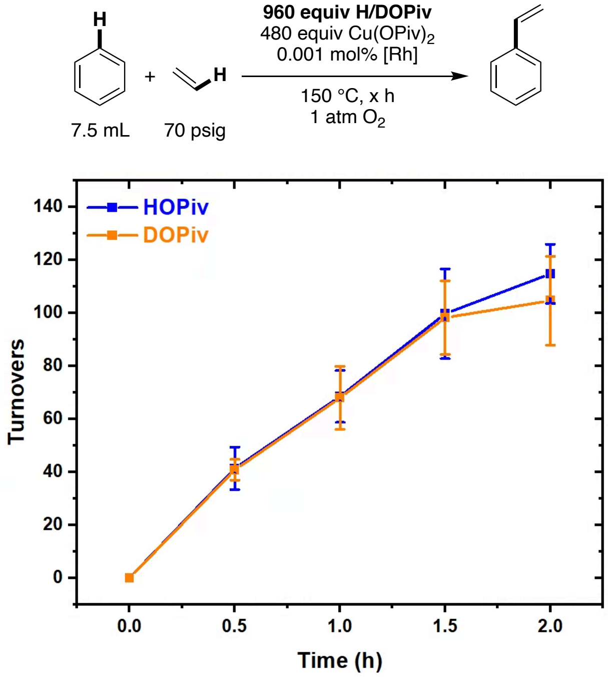

Figure S13. Turnovers versus time plot for benzene ethenylation reactions in the presence of either HOPiv or DOPiv using $Cu(OPiv)_2$ as the oxidant at aerobic conditions. Reaction conditions: 7.5 mL benzene, 0.001 mol% (based on single Rh atom) $[(\eta^2\text{-}C_2H_4)_2Rh(\mu\text{-}OAc)]_2$, 480 equiv (based on single Rh atom) $Cu(OPiv)_2$, 960 equiv HOPiv or

DOPiv, 70 psig ethylene, 1 atm O_2, 150 °C. Each data point represents the average from a minimum of three independent experiments and the error bars represent the standard deviation from the multiple experiments.

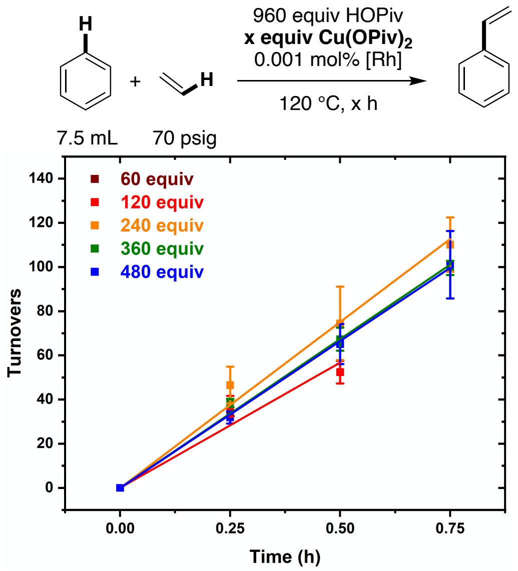

Figure S14. Turnovers versus time plot for benzene ethenylation reactions at varying $Cu(OPiv)_2$ concentration using $Cu(OPiv)_2$ as the oxidant at anaerobic conditions. Reaction conditions: 7.5 mL benzene, 0.001 mol% (based on single Rh atom) $[(\eta^2\text{-}C_2H_4)_2Rh(\mu\text{-}OAc)]_2$, x equiv (based on single Rh atom) $Cu(OPiv)_2$, 960 equiv HOPiv, 70 psig ethylene, 120 °C. Each data point represents the average from a minimum of three independent experiments and the error bars represent the standard deviation from the multiple experiments.

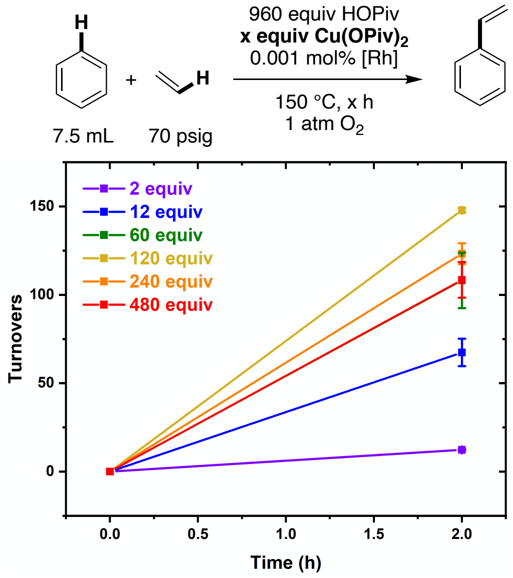

Figure S15. Turnovers versus time plot for benzene ethenylation reactions at varying $Cu(OPiv)_2$ concentration using $Cu(OPiv)_2$ as the oxidant at aerobic conditions. Reaction conditions: 7.5 mL benzene, 0.001 mol% (based on single Rh atom) $[(\eta^2\text{-}C_2H_4)_2Rh(\mu\text{-}OAc)]_2$, x equiv (based on single Rh atom) $Cu(OPiv)_2$, 960 equiv HOPiv, 70 psig ethylene, 1 atm dioxygen, 150 °C. Each data point represents the average from a minimum of three independent experiments and the error bars represent the standard deviation from the multiple experiments.

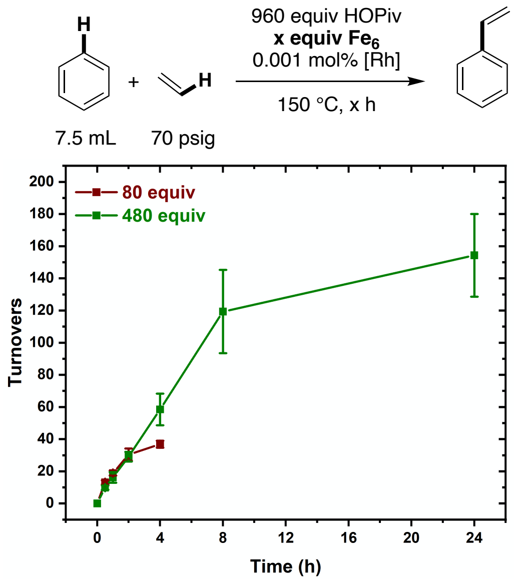

Figure S16. Turnovers versus time plot for benzene ethenylation reactions at varying $Fe_6(\mu\text{-}OH)_2(\mu_3\text{-}O)_2(\mu\text{-}OPiv)_{12}(\mu\text{-}HOPiv)_2$ loading. Reaction conditions: 7.5 mL benzene, 0.001 mol% (based on single Rh atom) $[(\eta^2\text{-}C_2H_4)_2Rh(\mu\text{-}OAc)]_2$, x equiv (based on single Rh atom) $Fe_6(\mu\text{-}OH)_2(\mu_3\text{-}O)_2(\mu\text{-}OPiv)_{12}(\mu\text{-}HOPiv)_2$, 960 equiv HOPiv, 70 psig ethylene, 150 °C. Each data point represents the average from a minimum of three independent experiments and the error bars represent the standard deviation from the multiple experiments. **Fe_6** = $Fe_6(\mu\text{-}OH)_2(\mu_3\text{-}O)_2(\mu\text{-}OPiv)_{12}(\mu\text{-}HOPiv)_2$.
